# Synthesis of a heparan sulfate tetrasaccharide using automated glycan assembly†

**DOI:** 10.1039/d3ob01909h

**Published:** 2024-01-24

**Authors:** Imlirenla Pongener, Eric T. Sletten, José Danglad-Flores, Peter H. Seeberger, Gavin J. Miller

**Affiliations:** a School of Chemical and Physical Sciences & Centre for Glycoscience, Keele University Keele Staffordshire ST5 5BG UK g.j.miller@keele.ac.uk; b Department of Biomolecular Systems, Max-Planck Institute of Colloids and Interfaces Am Mühlenberg 1 14476 Potsdam Germany

## Abstract

Herein we utilise automated glycan assembly to complete solid-phase synthesis of defined heparan sulfate oligosaccharides, employing challenging d-glucuronate disaccharide donors. Using an orthogonally protected d-GlcN-α-d-GlcA donor, milligram-scale synthesis of a heparan sulfate tetrasaccharide is completed in 18% yield over five steps. Furthermore, orthogonal protecting groups enabled regiospecific on-resin 6-*O*-sulfation. This methodology provides an important benchmark for the rapid assembly of biologically relevant heparan sulfate sequences.

## Abstract 2

Herein we utilise automated glycan assembly to complete solid-phase synthesis of defined heparan sulfate oligosaccharides, employing on resin O-sulfation and d-glucuronate disaccharide donors.

## Graphical abstract

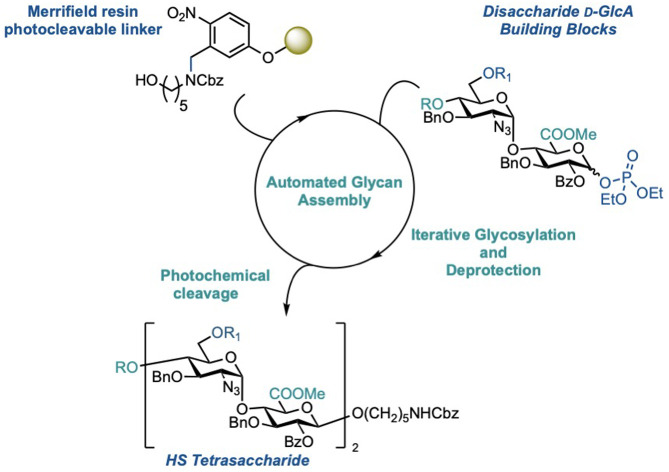

Heparan sulfate (HS) is a linear, highly sulfated glycosaminoglycan (GAG) present on most animal cell surfaces and in the surrounding extracellular matrix (Fig. 1a). This ubiquitous polysaccharide mediates mammalian cell function alongside pathological conditions, including cancer, Alzheimer's disease and viral infections.^1–5^ Accordingly, efficient syntheses of defined HS fragments,^3,6–13^ are key to provide materials for establishing structure–function relationships and to explore new therapeutic opportunities.^14–18^ Extensive work utilising solution-phase chemical synthesis to access an exciting array of HS targets has been reported.^19–22^ However, there are comparatively few reports using a solid-phase synthesis approach to assemble HS or HS oligosaccharide precursors.^23–25^ To this end, Automated Glycan Assembly (AGA)^26^ is a powerful solid phase-based technology for the construction of homogeneous oligosaccharides and successful strategies implementing AGA have been reported to access simpler GAGs including chondroitin sulfate,^27^ dermatan sulfate,^28^ keratan sulfate,^29^ as well as an HS precursor (Fig. 1b).^30^ Herein we report the first example of HS tetrasaccharide synthesis using AGA, successfully employing a d-GlcN-α-d-GlcA disaccharide building block and glycosyl phosphate donors to effect diastereoselective glycosylation (Fig. 1c). This investigation provides a benchmark for the iterative solid-phase assembly of HS using AGA.

**Fig. 1:**
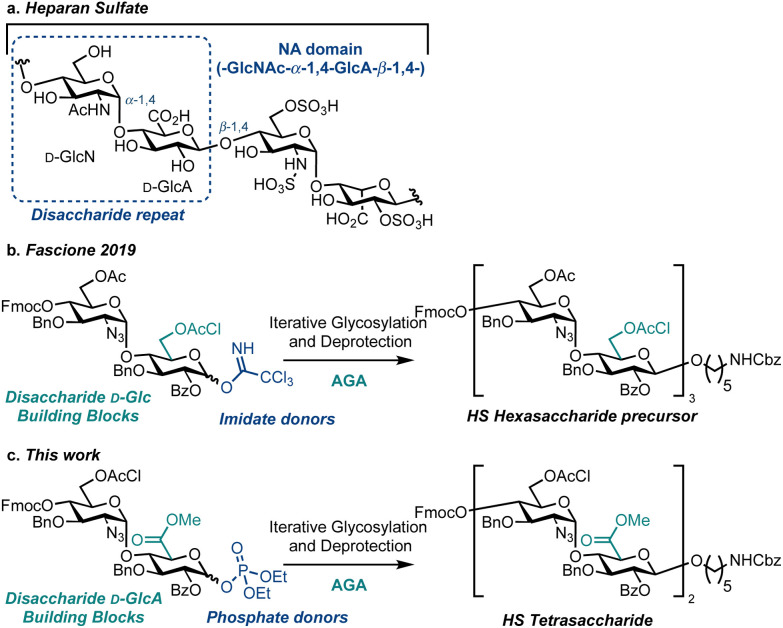
(a) The chemical structure of HS (NA domain shown in dotted blue box), broadly consisting of repeating disaccharide units of glucosamine (d-GlcN) and a uronic acid. (b) d-Glucose-based disaccharide donors for iterative assembly of an HS precursor. (c) Direct access to d-glucuronate oxidation level oligosaccharide sequences using d-GlcN-α-d-GlcA disaccharide phosphate donors.

At a macrostructural level, HS polysaccharides display distinct regions, conferring lower (NA) or higher (NS) levels of backbone *O*/*N*-sulfation and l-iduronic acid content. Homogenous synthetic oligosaccharides that mimic NA domains are valuable biochemical tools to explore domain function, and provide substrates to complete chemoenzymatic modifications, such as uronate C5 epimerisation and *O*/*N*-sulfation.^16,31^ To gain access to such materials, we focused our building block design on the disaccharide d-GlcN-α-d-GlcA, preinstalling the challenging d-GlcN-α-1,4 linkage internally, to prevent diastereoselectivity issues during the iterative [2 + 2] oligosaccharide assembly strategy. Accordingly, we placed a d-GlcA unit at the reducing end of the building block and used a methodology to prepare and employ this uronate donor in solution phase oligosaccharide synthesis (glycosyl β-phosphates resulted in glycosylation yields exceeding 60%).^20^

Prior to exploring iterative glycosylation using AGA whilst employing this building block, the optimal activation temperature for disaccharide phosphates 4–6 was established (Scheme 1). Different protecting groups were placed at the O-4′ and O-6′ positions of d-GlcN. The temporary protecting groups Lev and Fmoc were installed at O-4′ (to unmask an acceptor) for chain elongation and orthogonal TBDPS and AcCl groups at O-6′ (in anticipation of subsequent regiospecific sulfation). For the synthesis of glycosyl phosphates 4 and 5, a combination of Et_3_N and DMAP were used, while for phosphate 6, K_2_CO_3_ and Cs_2_CO_3_ were used in the presence of a labile Fmoc group. The O-2 position of d-GlcA was masked with a benzoyl ester to direct the formation of the β-1,4 linkage during iterative glycosylations.

**Scheme 1:**
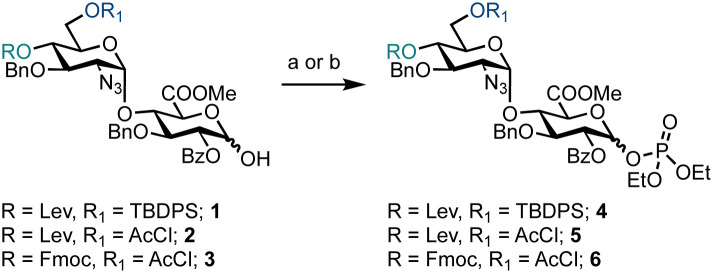
Synthesis of differentially protected HS disaccharide phosphate building blocks 4–6, reagents and conditions: (a) ClP(O)(OEt)_2_, Et_3_N, DMAP, CH_2_Cl_2_, 0 °C, 4 = 93%, α/β = 27 : 73, 5 = 88%, α/β = 26 : 74; (b) ClP(O)(OEt)_2_, K_2_CO_3_, Cs_2_CO_3_, CH_2_Cl_2_, 0 °C to rt, 6 = 84%, α/β = 3 : 97.

Semi-automated temperature-controlled experiments aid in selecting reaction temperatures for thioglycoside building blocks to be used in AGA synthesisers.^32,33^ Identification of the optimal reaction temperature drastically improves the efficiency of AGA processes, as illustrated in the context of a β-(1–4) glucan synthesis.^32^ Activation experiments with glycosyl phosphates, in the absence of a nucleophile, at different temperatures were carried out in the AGA reactor (Table 1).^28^ A solution of the building block in CH_2_Cl_2_ was cooled to the required temperature and treated with a solution of TMSOTf in CH_2_Cl_2_. Mixing was enabled *via* argon bubbling for five minutes at the set temperature, before neutralisation with pyridine/DMF and ejection from the reactor to the fraction collector. Following aqueous work-up, the crude material was analysed by ^1^H and ^31^P NMR spectroscopy for the presence of remaining glycosyl phosphate and/or hemi-acetal hydrolysis product (1–3).

**Table 1:** Temperature activation studies of glycosyl phosphate building blocks 4–6 that contain GlcA at the reducing end

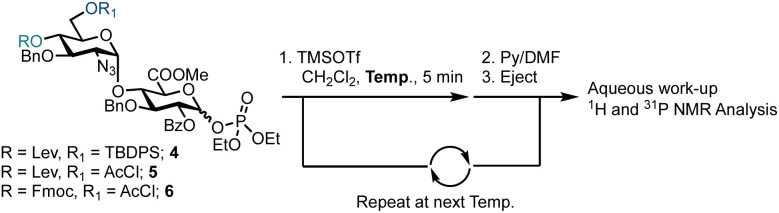						
Entry	Glycosyl phosphate				Temp. (°C)	Observation
		R	R_1_	α/β		
1	4	Lev	TBDPS	48 : 52	−35	α-4 only
2					−20	α-4 only
3					0	α-4 only
4	5	Lev	AcCl	32 : 68	−20	α/β 33 : 67, no activation
5					0	α/β 36 : 64, ∼5% activation
6	6	Fmoc	AcCl	22 : 78	−20	α-6 only
7					0	α-6 only

For building block 4 (6′-OTBDPS/4′-OLev), the β-glycosyl phosphate was activated at −35 °C, such that only α-4 remained (Table 1, entry 1). The same result was observed at −20 °C and 0 °C (Table 1, entries 2 and 3). It is not clear whether the building block was activated and the anomeric leaving group lost, followed by formation of a thermodynamically favoured α-phosphate or *in situ* anomerisation occurred under the activation conditions. Furthermore, α-4 may be unreactive under the conditions screened. Building block 5 (6′-OAcCl/4′-OLev) was unreactive (Table 1, entries 4 and 5) as only 5% of 2 was activated at 0 °C and no reactivity observed at −20 °C, likely due to a combined disarming effect of the AcCl and Lev esters.^34^ Building block 6 (6′-OAcCl/4′-OFmoc), much like 4, was activated at −20 °C, with only α-6 observed by NMR (Table 1, entries 6 and 7). Indicatively, based on remote protecting group electronics, replacement of a 4′-OLev ester with 4′-OFmoc carbonate improved the building block reactivity similarly to the replacement of 6′-OAcCl ester by a 6′-OTBDPS ether. Subsequently, building block 4 was employed for AGA.

Disaccharide glycosyl phosphate building block 4 (4.5 eq.) was tested to glycosylate aminopentanol photocleavable linker 7 by AGA (Scheme 2). Glycosylations using 4 and TMSOTf as an activator at −35 °C for 20 minutes and then at −5 °C for 10 minutes were completed. Capping by acetylating any unreacted nucleophile 7 was followed by 4′-OLev cleavage with hydrazine in acetic acid. This coupling cycle was repeated to prepare tetrasaccharide 8. Analysis of the reactions by MALDI, following micro-photocleavage from the solid support revealed that the 6′-OTBDPS group was cleaved during the acidic capping step (Ac_2_O, MsOH in CH_2_Cl_2_, 25 °C for 20 minutes). The 6′-hydroxyl group thereby liberated was acetylated such that subsequent glycosylation using 4 formed a tetrasaccharide with a mixed d-GlcN-*O*-6 protecting group pattern (6′-OAc and 6′′′-OTBDPS). Exclusion of the capping step from the automation sequence to avoid TBDPS cleavage, and glycosylation with six eq. of 4 and longer reaction times (40 minutes *versus* 20 minutes) showed that both glycosylation and removal of the temporary Lev protecting group were successful, but no tetrasaccharide had been formed. The bulky 6′-OTBDPS close to the nucleophile may have hindered subsequent glycosylation.

**Scheme 2:**
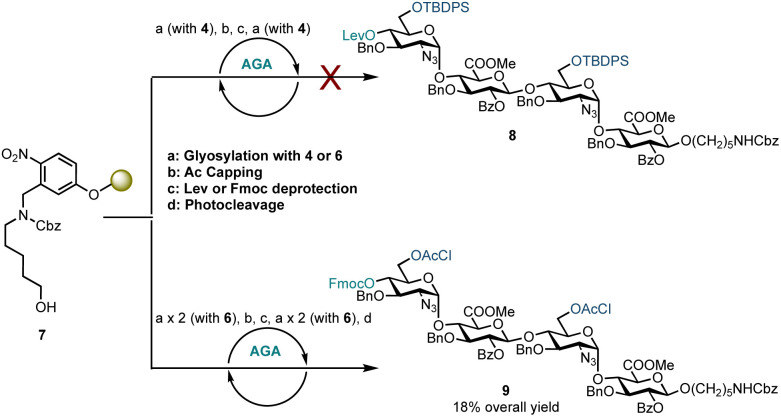
AGA of HS tetrasaccharides using d-GlcN-α-d-GlcA disaccharide building blocks. Conditions for the use of building block 4: a: 4.5 eq. building block (0.0653 mmol), TMSOTf, temp.: *T*_1_ = −35 °C for 20 min, *T*_2_ = −5 °C for 10 min; b: Ac_2_O, MsOH, CH_2_Cl_2_, temp.: 25 °C for 20 min; c: N_2_H_4_·AcOH, pyridine/AcOH/H_2_O, temp.: 40 °C for 5 min × 3. Conditions for the use of donor 6: a: 4.5 eq. donor (0.0653 mmol), TMSOTf, temp.: *T*_1_ = −15 °C for 35 min, *T*_2_ = 0 °C for 35 min; b: Ac_2_O, MsOH, CH_2_Cl_2_, temp.: 30 °C for 10 min; c: 5% Et_3_N in DMF, temp.: 25 °C for 5 min; d: temp.: rt, 14 h, UV = 370 nm.

Based on these observations, disaccharide glycosyl phosphate building block 6 (6′-OAcCl/4′-OFmoc, Scheme 2) was evaluated next. The AGA coupling cycle was modified such that the glycosylation reaction was performed twice at reactor temperatures between −15 and 0 °C, to promote high glycosylation yields.^27,30^ Any unreacted aminopentanol linker 7 was capped and the 4′-OFmoc group removed using triethylamine in DMF to unmask a new disaccharide acceptor for a [2 + 2] glycosylation with 6. Excess building block 6 was retrieved in hemi-acetal form in the fraction collector to be recycled for subsequent syntheses. Pleasingly, following a second glycosylation sequence, the desired tetrasaccharide 9 was cleaved from the resin using UV light (370 nm). HPLC analysis of the crude product indicated 9 to be the major product with very little deletion sequence being present (see ESI† for further details). Preparative HPLC produced 5.0 mg of target tetrasaccharide 9 in 18% yield over five steps. Tetrasaccharide product 9 was confirmed by ESI-HRMS and NMR analysis (Fig. 2). Installation of β-linkages was identified through ^1^*J*_C–H_ coupling constants (^1^*J*_C1−H1_ = 165 Hz and ^1^*J*_C1′′–H1′′_ = 167 Hz).

**Fig. 2:**
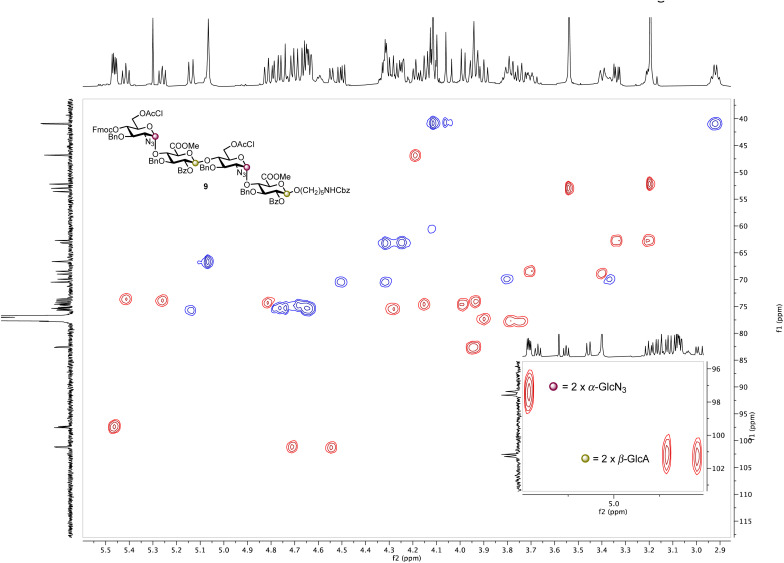
HSQC NMR spectrum of tetrasaccharide 9 (600 MHz × 151 MHz, chloroform-*d*) highlighting the presence of two α-linkages (purple dots) and two β-linkages (yellow dots).

With a method for HS oligosaccharide assembly in place we investigated on-resin sulfation. Solid-supported sulfation reactions are advantageous as they curtail the challenges often encountered during batch handling of highly polar compounds including reaction work-up and purification. On-resin sulfation had been used in the synthesis of chondroitin sulfate,^27^ dermatan sulfate,^28^ keratan sulfate,^29,35^ and other sulfated glycans.^36^ In order to explore the potential for on-resin sulfation of our HS sequence, we synthesised and isolated sulfated tetrasaccharide 10 using building block 6 (Scheme 3). Following tetrasaccharide backbone assembly, the 6′-OAcCl and 6′′′-OAcCl groups were selectively removed using thiourea in 2-methoxyethanol and pyridine. The free hydroxyl groups were then reacted with sulfur trioxide trimethylamine complex in DMF at 90 °C. Reverse-phase LCMS analysis of the crude material indicated formation of 10 along with the disaccharide deletion sequence. Sulfated tetrasaccharide 10 was purified by preparative HPLC to obtain 1.0 mg of the desired product, corresponding to 3% yield over eight steps. However, this material degraded during NMR analysis in CDCl_3_.

**Scheme 3:**
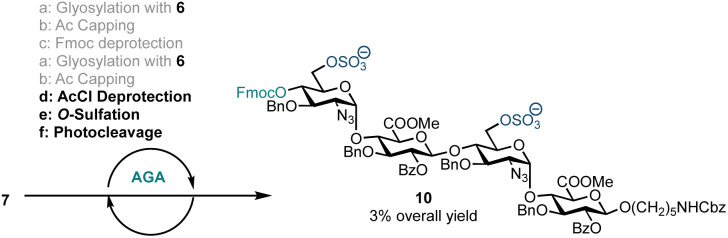
AGA including *O*-sulfation of an HS tetrasaccharide using disaccharide building block 6. a: 4.5 eq. building block (0.0653 mmol), TMSOTf, temp.: *T*_1_ = −15 °C for 35 min, *T*_2_ = 0 °C for 35 min; b: Ac_2_O, MsOH, CH_2_Cl_2_, temp.: 25 °C for 5 min; c: 20% Et_3_N in DMF, temp.: 25 °C for 5 min; d: thiourea, 2-methoxyethanol, pyridine, temp.: 80 °C for 20 min; e: SO_3_·Me_3_N, DMF (0.4 M), temp.: 90 °C for 30 min; f: temp.: rt, 14 h, UV = 370 nm.

In conclusion, we have completed the first synthesis of an orthogonally protected HS tetrasaccharide using AGA and a d-GlcN-α-d-GlcA disaccharide glycosyl phosphate building block. An orthogonal protecting group system relying on 6′-OAcCl and 4′-OFmoc was established; the best reaction temperature was determined for iterative glycosylation using disaccharides allowing for regiospecific d-GlcN 6-*O*-sulfation. AGA using disaccharide glycosyl phosphates delivered a diastereomerically pure tetrasaccharide sequence equipped with a conjugation-ready, reducing end tether. Regiospecific on-resin 6-*O*-sulfation by AGA completed the synthesis as an important blueprint to enable the synthesis of bespoke and biologically relevant NA domain HS sequences using AGA, without the need of post-glycosylation oxidation to access the uronate component.

## Conflicts of interest

There are no conflicts to declare.

## Supplementary Material

OB-022-D3OB01909H-s001
