# Supplementary material for: Synthesis of a heparan sulfate tetrasaccharide using automated glycan assembly
Source: Org Biomol Chem. 2024 Jan 24;22(7):1395–9. doi: 10.1039/d3ob01909h (PMC10865181; doi:10.1039/d3ob01909h)
Supplement: OB-022-D3OB01909H-s001 [file OB-022-D3OB01909H-s001.pdf]

Electronic Supplementary Material (ESI) for Organic & Biomolecular Chemistry.
This journal is © The Royal Society of Chemistry 2024

Automated Solid-Phase Synthesis of a Heparan Sulfate Tetrasaccharide

Imlirenla Pongener,[a] Eric T. Sletten,[b] José Danglad-Flores,[b] Peter H. Seeberger[b] and Gavin J. Miller[a]*

[a]School of Chemical and Physical Sciences & Centre for Glycoscience, Keele University, Keele, Staffordshire, ST5 5BG, United Kingdom
[b]Department of Biomolecular Systems, Max-Planck Institute of Colloids and Interfaces Am Mühlenberg 1, 14476 Potsdam, Germany

*Email: g.j.miller@keele.ac.uk

Table of Contents

1. General Experimental 2
2. Synthesis of Building Blocks for AGA 3
    2.1 Synthesis of 2 3
    2.2 Synthesis of 4 4
    2.3 Synthesis of 5 5
    2.4 Synthesis of 6 7
3. Automated Synthesis 8
    3.1 Materials and Measurements 8
    3.2 Preparation of Stock Solutions 8
    3.3 Modules for Automated Synthesis 9
    3.4 Synthesis of 9 11
    3.5 Synthesis of 10 13
4. Spectra 16
    4.1 Compound 2 NMR 16
    4.2 Compound 4 NMR 18
    4.3 Compound 5 NMR 21
    4.4 Compound 6 NMR 24
    4.5 Compound 9 NMR 28
    4.6 Compound 10 NMR 31
5. References 31

1. General Experimental

The reagents and solvents used in the following experiments were bought commercially and used without further purification. Anhydrous solvents were obtained using equipment based on Grubb's design[1] and stored under N_2 in Young's flask over 4 Å molecular sieves. Anhydrous DMF and Pyridine were purchased from Acros. For air-sensitive reactions, solvents were added *via* syringe through rubber septa. Reactions were monitored by thin layer chromatography using Merck silica-coated 60F254 aluminium plates and the eluents are outlined in the respective experiments; spots were detected under 254 nm UV light and 10% H_2SO_4/EtOH staining followed by heat. Flash column chromatography was performed using silica gel [Davisil, 400–230 mesh (63–40 µm)]. Analysis and purification by normal and reverse phase HPLC were performed using the Agilent 1260 series equipped with a Multiple Wavelength Detector (MWD) and an Evaporative Light Scattering Detector (ELSD). ^{1}H NMR, ^{13}C NMR and 2D NMR were carried out at 400 MHz on a Bruker AVIII400 or on a Varian 600-MR (600 MHz) spectrometer using deuterated chloroform ($CDCl_3$). Chemical shifts are reported in parts per million (ppm), coupling constants (*J*) are reported in Hertz (Hz) and multiplicities are abbreviated as; s (singlet), d (doublet), t (triplet) or m (multiplet) or combinations thereof. Chemical shifts were referenced to the residual proton of TMS for ^{1}H NMR spectra and to the ^{13}C chemical shift of deuterated chloroform ($CDCl_3$) for ^{13}C NMR spectra. For compounds not reported in literature, NMR assignments have been made using COSY, HSQC and HMBC. HRMS were recorded on a ThermoScientific LTQ Orbitrap XL at the ESPRC National Mass Spectrometry Facility at Swansea University, 6210 ESI-TOF mass spectrometer (Agilent) and MALDI-TOF autoflexTM (Bruker) instruments. The automated syntheses were performed on an automated solid phase synthesiser developed at the Max Planck Institute of Colloids and Interfaces.

2. Synthesis of Building Blocks for AGA

Synthesis and characterisation of ***S1, 1*** *and* ***3*** *have been reported previously.*[2]

2.1 Methyl (2-azido-3-*O*-benzyl-6-*O*-chloroacetyl-2-deoxy-4-*O*-levulinoyl-α-D-glucopyranosyl)-(1→4)-(2-*O*-benzoyl-3-*O*-benzyl-α/β-D-glucopyranosyluronate) 2

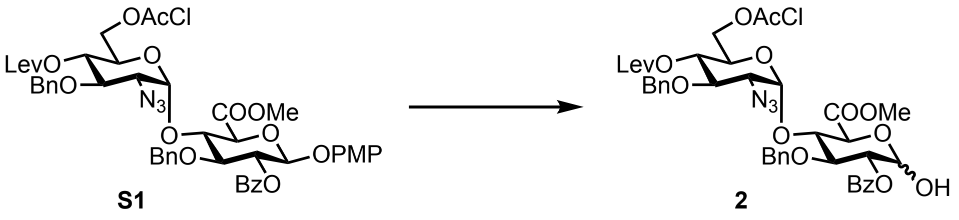

A solution of disaccharide **S1** (757 mg, 0.788 mmol) in Tol/MeCN/H_2O (1:1.5:1; 16 mL) was cooled to 0 °C and treated with CAN (2.16 g, 3.94 mmol). The reaction mixture was then stirred at room temperature for 1 h. The reaction was diluted with EtOAc (25 mL) and washed with H_2O (50 mL). The layers were separated, and the product was extracted with EtOAc (3 x 25 mL). The combined organic layers were dried over anhydrous $MgSO_4$, filtered and concentrated *in vacuo* to give a brown paste. Purification by column chromatography (R_f = 0.58, 1:1; hex/EtOAc) gave hemiacetal **2** as a light brown foam (622 mg, 92% yield, α/β = 88:12).

α-anomer

^{1}H NMR (400 MHz, $CDCl_3$): δ 8.13 – 8.07 (m, 2H, Ph), 7.61 – 7.52 (m, 1H, Ph), 7.46 (t, *J* = 7.7 Hz, 2H, Ph), 7.42 – 7.13 (m, 10H, Ph), 5.65 (dd, *J* = 5.8, 3.2 Hz, 1H, H-1), 5.44 (d, *J* = 3.7 Hz, 1H, H-1'), 5.13 (dd, *J* = 8.1, 3.1 Hz, 1H, H-2), 4.96 (dd, *J* = 10.4, 9.2 Hz, 1H, H-4'), 4.89 – 4.81 (m, 2H, 2 x C*H*HPh), 4.70 (d, *J* = 7.8 Hz, 1H, H-5), 4.60 (d, *J* = 11.1 Hz, 1H), 4.49 (d, *J* = 11.1 Hz, 1H), 4.38 (t, *J* = 7.7 Hz, 1H, H-3), 4.28 (dd, *J* = 12.3, 2.1 Hz, 1H, H-6a'), 4.25 – 4.10 (m, 4H, H-4, H-6b', AcCl-CH_2), 3.87 (ddd, *J* = 10.5, 5.2, 2.1 Hz, 1H, H-5'), 3.79 (dd, *J* = 10.2, 9.2 Hz, 1H, H-3'), 3.75 (s, 3H, OCH_3), 3.37 (d, *J* = 6.1 Hz, 1H, OH), 3.31 (dd, *J* = 10.3, 3.7 Hz, 1H, H-2'), 2.78 – 2.61 (m, 2H, Lev-CH_2), 2.53 (ddd, *J* = 17.2, 7.8, 5.5 Hz, 1H, Lev-C*H*H), 2.38 (dt, *J* = 17.2, 6.4 Hz, 1H, Lev-C*H*H), 2.16 (s, 3H, Lev-CH_3). **^{13}C{^{1}H} NMR** (101 MHz, $CDCl_3$): δ 206.4 (C=O), 171.9 (C=O), 169.6 (C=O), 167.6 (C=O), 165.8 (C=O), 137.6 (C), 137.51 (C), 133.7 (CH), 130.0 (CH), 129.5 (C), 128.8 (CH), 128.6 (2 x CH), 128.5 (2 x CH), 128.03 (CH), 127.98 (CH), 127.9 (CH), 127.7 (CH), 98.3 (C-1'), 90.2 (C-1), 77.9 (C-3), 75.7 (C-4), 75.0 (Ph*C*H_2), 74.8 (Ph*C*H_2), 72.8 (C-2), 71.2 (C-5), 70.4 (C-4'), 68.9 (C-5'), 63.7 (C-6'), 63.0 (C-2'), 52.9 (OCH_3), 41.2 (AcCl-*C*H_2), 37.9 (Lev-*C*H_2), 29.9 (Lev-*C*H_3), 28.0 (Lev-*C*H_2).

β-anomer

^{1}H NMR (400 MHz, $CDCl_3$) selected signals: δ 5.48 (d, *J* = 3.8 Hz, 1H, H-1'), 5.21 (dd, *J* = 8.3, 7.0 Hz, 1H, H-2), 4.04 (br s, 1H, OH), 3.79 (s, 3H, OCH_3), 3.33 (dd, *J* = 10.6, 3.7 Hz, 1H, H-2'). **^{13}C{^{1}H} NMR** (101 MHz, $CDCl_3$) selected signals: δ 171.8 (C=O), 169.2 (C=O), 167.4 (C=O), 166.7 (C=O), 137.53 (C), 137.2 (C), 133.9 (CH), 97.8 (C-1'), 96.0 (C-1), 81.0 (C-3), 75.3 (C-2), 63.5 (C-6'), 62.9 (C-2'), 53.2 (OCH_3), 41.1 (AcCl-CH_2).

ESI-HRMS for $C_{41}H_{44}O_{15}N_3ClNa$ $(M+Na)^+$ calculated: 876.2359; found: 876.2335.

2.2 Diethyl methyl (2-azido-3-*O*-benzyl-6-*O*-*tert*-butyldiphenylsilyl-2-deoxy-4-*O*-levulinoyl-α-D-glucopyranosyl)-(1→4)-(2-*O*-benzoyl-3-*O*-benzyl-α/β-D-glucopyranosyluronate) phosphate 4

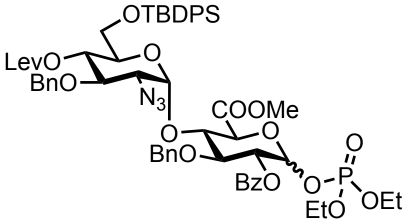

Under a N_2 atmosphere, a solution of hemiacetal **1** (208 mg, 0.205 mmol), Et_3N (0.17 mL, 1.2 mmol) and DMAP (2.6 mg, 0.021 mmol) in anhydrous CH_2Cl_2 (1 mL) was cooled to 0 °C and treated with diethyl chlorophosphate (0.06 mL, 0.4 mmol). The reaction was continued stirring at 0 °C. TLC analysis (90:10; CH_2Cl_2/EtOAc, R_f = 0.63) after 1 h showed complete consumption of starting material. The reaction mixture was directly purified by column chromatography (90:10; CH_2Cl_2/EtOAc with 0.5% Et_3N) to give phosphate **4** as a foam (220 mg, 93% yield, α/β = 27:73).

The following were observed for α and β anomers: **^{1}H NMR** (400 MHz, $CDCl_3$): δ 8.13 – 7.98 (m, 2H, Ph), 7.71 – 7.54 (m, 6H, Ph), 7.47 – 7.29 (m, 12H, Ph), 7.24 – 7.13 (m, 5H, Ph), 3.68 (dd, *J* = 11.7, 2.2 Hz, 1H, H-6a'), 3.63 (dd, *J* = 11.7, 3.0 Hz, 1H, H-6b'), 2.63 (t, *J* = 7.0 Hz, 2H, Lev-CH_2), 2.37 (td, *J* = 6.7, 2.0 Hz, 2H, Lev-CH_2), 2.14 (s, 3H, Lev-CH_3), 1.02 (s, 9H, $SiC(CH_3)_3$).

α-anomer

^{1}H NMR (400 MHz, $CDCl_3$) selected signals: δ 5.94 (dd, *J* = 7.1, 3.3 Hz, 1H, H-1), 5.35 (t, *J* = 9.7 Hz, 1H, H-4'), 5.24 (dt, *J* = 9.6, 3.0 Hz, 1H, H-2), 4.92 (d, *J* = 10.6 Hz, 1H, C*H*HPh), 4.87 (d, *J* = 10.6 Hz, 1H, C*H*HPh), 4.78 (d, *J* = 10.0 Hz, 1H, C*H*HPh), 4.65 (d, *J* = 11.0 Hz, 1H, C*H*HPh), 4.53 (d, *J* = 9.7 Hz, 1H, H-5), 4.33 (t, *J* = 9.1 Hz, 1H, H-3), 3.53 (s, 3H, OCH_3), 3.38 (dd, *J* = 11.0, 3.6 Hz, 1H, H-2'), 1.25 – 1.18 (m, 3H, Et-CH_3), 1.15 (td, *J* = 7.1, 1.0 Hz,

3H, Et-CH_3). **$^{13}C\{^1H\}$ NMR** (101 MHz, $CDCl_3$) selected signals from HSQC: δ 93.8 (C-1), 78.8 (c-3), 72.5 (C-2), 72.0 (C-5), 63.1 (C-2'). **$^{31}P\{^1H\}$ NMR** (162 MHz, $CDCl_3$): δ -2.75.

β-anomer

^1H NMR (400 MHz, $CDCl_3$): δ 5.52 – 5.42 (m, 3H, H-1, H-1', H-4'), 5.34 (t, J = 9.6 Hz, 1H, H-2), 4.81 (d, J = 10.5 Hz, 1H, C*H*HPh), 4.79 (d, J = 11.0 Hz, 1H, C*H*HPh), 4.72 (d, J = 10.5 Hz, 1H, C*H*HPh), 4.66 (d, J = 11.0 Hz, 1H, C*H*HPh), 4.28 – 4.18 (m, 2H, H-4, H-5), 4.13 – 3.98 (m, 3H, Et-OCH_2, H-3), 3.90 (dd, J = 10.4, 9.2 Hz, 1H, H-3'), 3.86 – 3.73 (m, 2H, Et-OCH_2), 3.56 (s, 3H, OCH_3), 3.53 – 3.47 (m, 1H, H-5'), 3.35 (dd, J = 10.4, 3.6 Hz, 1H, H-2'), 1.25 (td, J = 7.1, 1.1 Hz, 3H, Et-CH_3), 0.95 (td, J = 7.1, 1.1 Hz, 3H, Et-CH_3). **$^{13}C\{^1H\}$ NMR** (101 MHz, $CDCl_3$): δ 206.3 (C=O), 171.1 (C=O), 167.9 (C=O), 165.1 (C=O), 137.7 (C), 137.1 (C), 135.92 (CH), 135.87 (CH), 133.8 (CH), 133.4 (C), 133.3 (C), 130.0 (CH), 129.8 (CH), 129.7 (CH), 129.2 (C), 128.7 (CH), 128.6 (2 x CH), 128.3 (CH), 128.1 (CH), 128.0 (CH), 127.73 (CH), 127.72 (CH), 97.6 (C-1'), 96.4 (d, J = 4.8 Hz, C-1), 82.0 (C-3), 77.7 (C-3'), 74.9 (C-5), 74.8 (Ph*C*H_2), 74.5 (Ph*C*H_2), 74.1 (C-4), 73.0 (d, J = 8.5 Hz, C-2), 71.2 (C-5'), 69.9 (C-4'), 64.7 (d, J = 6.0 Hz, Et-O*C*H_2), 64.4 (d, J = 6.2 Hz, Et-O*C*H_2), 63.0 (C-2'), 61.6 (C-6'), 52.7 (OCH_3), 37.9 (Lev-*C*H_2), 30.0 (Lev-*C*H_3), 28.0 (Lev-*C*H_2), 26.9 (SiC(*C*H_3)$_3$), 19.4 (Si*C*(CH_3)$_3$), 15.9 (d, J = 7.3 Hz, Et-*C*H_3), 15.7 (d, J = 7.2 Hz, Et-*C*H_3). **$^{31}P\{H\}$ NMR** (162 MHz, $CDCl_3$): δ -3.28 (β).

ESI-HRMS for $C_{59}H_{70}O_{17}N_3SiPNa$ $(M+Na)^+$ calculated: 1174.4110; found: 1174.4082.

2.3 Diethyl methyl (2-azido-3-*O*-benzyl-6-*O*-chloroacetyl-2-deoxy-4-*O*-levulinoyl-α-D-glucopyranosyl)-(1→4)-(2-*O*-benzoyl-3-*O*-benzyl-β-D-glucopyranosyluronate) phosphate 5

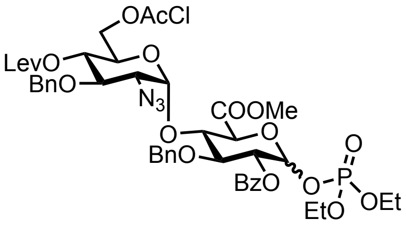

Under a N_2 atmosphere, a solution of hemiacetal **2** (200 mg, 0.234 mmol), Et_3N (0.20 mL, 1.4 mmol) and DMAP (2.8 mg, 0.023 mmol) in anhydrous CH_2Cl_2 (1 mL) was cooled to 0 °C and treated with diethyl chlorophosphate (0.07 mL, 0.5 mmol). The reaction was continued stirring at 0 °C. TLC analysis (R_f = 0.48, 1:1; hex/EtOAc) after 1 h showed complete consumption of starting material. The reaction mixture was directly purified by column chromatography (1:1;

hex/EtOAc with 0.5% Et_3N) to give phosphate **5** as a light brown syrup (205 mg, 88% yield, α/β = 26:74).

The following were observed for α and β anomers: **^{1}H NMR** (400 MHz, $CDCl_3$): δ 8.08 – 7.98 (m, 2H, Ph), 7.63 – 7.54 (m, 1H, Ph), 7.50 – 7.40 (m, 2H, Ph), 7.37 – 7.28 (m, 5H, Ph), 7.23 – 7.11 (m, 5H,Ph), 4.25 – 4.19 (m, 2H, H-6a', H-6b'), 4.19 – 4.02 (m, 4H, AcCl-CH_2, Et-OCH_2), 3.85 – 3.74 (m, 2H, Et-OCH_2), 3.80 (s, 3H, OCH_3), 3.67 (dt, *J* = 10.4, 3.3 Hz, 1H, H-5'), 2.78 – 2.60 (m, 2H, Lev-CH_2), 2.50 (ddd, *J* = 17.2, 7.6, 5.4 Hz, 1H, Lev-C*H*H), 2.37 (ddd, *J* = 17.2, 6.7, 5.8 Hz, 1H, Lev-C*H*H), 2.16 (s, 3H, Lev-CH_3). **^{13}C{^{1}H} NMR** (101 MHz, $CDCl_3$): δ 171.8 (C=O), 68.9 (C-5'), 63.3 (C-6'), 41.1 (AcCl-*C*H_2), 37.9 (Lev-*C*H_2), 27.9 (Lev-*C*H_2).

α-anomer

^{1}H NMR (400 MHz, $CDCl_3$) selected signals: δ 5.94 (dd, *J* = 7.1, 3.3 Hz, 1H, H-1), 5.55 (d, *J* = 3.8 Hz, 1H, H-1'), 5.23 (dt, *J* = 9.6, 3.1 Hz, 1H, H-2), 5.05 – 4.95 (m, 1H, H-4'), 4.90 (d, *J* = 10.6 Hz, 1H, C*H*HPh), 4.86 (d, *J* = 10.6 Hz, 1H, C*H*HPh), 4.53 (d, *J* = 9.7 Hz, 1H, H-5), 4.38 – 4.29 (m, 1H, H-3), 4.27 – 4.21 (m, 1H, H-4), 3.41 – 3.36 (m, 1H, H-2'), 1.24 (td, *J* = 7.1, 1.0 Hz, 3H, Et-CH_3), 1.14 (td, *J* = 7.1, 1.1 Hz, 3H, Et-CH_3). **^{13}C{^{1}H} NMR** (101 MHz, $CDCl_3$) selected signals: δ 206.37 (C=O), 168.6 (C=O), 167.44 (C=O), 165.5 (C=O), 137.51 (C), 137.4 (C), 133.8 (CH), 129.9 (CH), 129.0 (C), 128.7 (CH), 128.2 (CH), 128.13 (CH), 128.07 (CH), 127.6 (CH), 97.9 (C-1'), 93.9 (d, *J* = 5.9 Hz, C-1), 78.9 (C-3), 75.5 (Ph*C*H_2), 75.19 (Ph*C*H_2), 75.1 (C-4), 72.8 (d, *J* = 7.7 Hz, C-2), 71.6 (C-5), 70.11 (C-4'), 64.5 (d, *J* = 5.6 Hz, Et-O*C*H_2), 63.0 (C-2'), 53.2 (OCH_3), 29.86 (Lev-*C*H_3), 16.1 (d, *J* = 6.8 Hz, Et-*C*H_3), 15.9 (d, *J* = 7.0 Hz, Et-*C*H_3). **^{31}P{^{1}H} NMR** (162 MHz, $CDCl_3$): δ -2.82.

β-anomer

^{1}H NMR (400 MHz, $CDCl_3$): δ 5.52 (d, *J* = 3.9 Hz, 1H, H1'), 5.50 (t, *J* = 7.3 Hz, 1H, H-1), 5.47 – 5.40 (m, 1H, H-2), 5.00 (dd, *J* = 10.4, 9.1 Hz, 1H, H-4'), 4.82 – 4.65 (m, 4H, 4 x C*H*HPh), 4.32 – 4.26 (m, 1H, H-4), 4.23 – 4.17 (m, 1H, H-5), 4.02 (t, *J* = 8.3 Hz, 1H, H-3), 3.89 (dd, *J* = 10.3, 9.0 Hz, 1H, H-3'), 3.34 (dd, *J* = 10.4, 3.8 Hz, 1H, H-2'), 1.29 (td, *J* = 7.1, 1.2 Hz, 3H, Et-CH_3), 0.96 (td, *J* = 7.1, 1.2 Hz, 3H, Et-CH_3). **^{13}C{^{1}H} NMR** (101 MHz, $CDCl_3$): δ 206.35 (C=O), 168.1 (C=O), 167.41 (C=O), 165.0 (C=O), 137.48 (C), 137.0 (C), 133.9 (CH), 130.0 (CH), 129.1 (C), 128.8 (CH), 128.6 (2 x CH), 128.5 (2 x CH), 128.10 (2 x CH), 127.9 (2 x CH), 97.6 (C-1'), 96.4 (d, *J* = 4.9 Hz, C-1), 82.1 (C-3), 77.3 (C-3'), 75.2 (Ph*C*H_2), 74.9 (Ph*C*H_2), 74.7 (C-4), 74.5 (C-5), 73.2 (d, *J* = 8.4 Hz, C-2), 70.14 (C-4'), 64.7 (d, *J* = 6.2 Hz, Et-O*C*H_2), 64.5 (d, *J* = 6.2 Hz, Et-O*C*H_2), 62.8 (C-2'), 53.1 (OCH_3), 29.87 (Lev-*C*H_3), 16.0 (d, *J* = 7.3 Hz, Et-*C*H_3), 15.7 (d, *J* = 7.0 Hz, Et-*C*H_3). **^{31}P{^{1}H} NMR** (162 MHz, $CDCl_3$): δ -3.28.

ESI-HRMS for $C_{45}H_{53}O_{18}N_3ClPNa$ $(M+Na)^+$ calculated: 1012.2648; found: 1012.2620

2.4 Diethyl methyl (2-azido-3-*O*-benzyl-6-*O*-chloroacetyl-2-deoxy-4-*O*-fluorenylmethoxycarbonyl-α-D-glucopyranosyl)-(1→4)-(2-*O*-benzoyl-3-*O*-benzyl-β-D-glucopyranosyluronate) phosphate 6

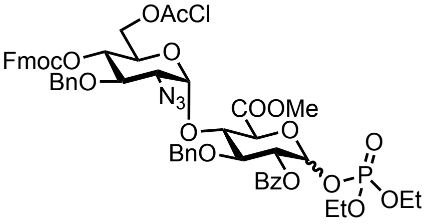

Under a N_2 atmosphere, a mixture of hemiacetal **3** (395 mg, 0.404 mmol) and K_2CO_3 (112 mg, 0.808 mmol) in anhydrous CH_2Cl_2 (1.3 mL) was treated with diethyl chlorophosphate (0.07 mL, 0.81 mmol) at 0 °C. After 5 minutes Cs_2CO_3 (132 mg, 0.404 mmol) was added to the reaction mixture. The reaction was then stirred at room temperature for 2.5 h. TLC analysis (1:1; hex/EtOAc, R_f = 0.5) showed complete consumption of starting material. The reaction mixture was directly purified by column chromatography (1:1; hex/EtOAc with 0.5% Et_3N) to give phosphate **6** as a white foam (380 mg, 84% yield, α/β = 3:97). **^{1}H NMR** (400 MHz, $CDCl_3$): δ 8.08 – 8.03 (m, 2H, Ph), 7.80 – 7.71 (m, 2H, Ph), 7.63 – 7.53 (m, 3H, Ph), 7.48 – 7.42 (m, 2H, Ph), 7.44 – 7.35 (m, 2H, Ph), 7.31 – 7.26 (m, 2H, Ph), 7.25 – 7.13 (m, 10H, Ph), 5.53 – 5.48 (m, 2H, H-1, H-1'), 5.44 (dd, *J* = 8.3, 7.4 Hz, 1H, H-2), 4.81 (dd, *J* = 10.3, 9.3 Hz, 1H, H-4'), 4.79 – 4.69 (m, 3H, 3 x C*H*HPh), 4.64 (d, *J* = 10.9 Hz, 1H, C*H*HPh), 4.51 (dd, *J* = 10.5, 6.6 Hz, 1H, Fmoc-CH*H*), 4.36 – 4.28 (m, 2H, Fmoc-CH*H*, H-4), 4.26 (app d, *J* = 3.3 Hz, 2H, H-6a', H-6b'), 4.23 – 4.16 (m, 1H, Fmoc-CH), 4.20 (d, *J* = 9.4 Hz, 1H, H-5), 4.16 – 4.06 (m, 4H, AcCl-CH_2, Et-OCH_2), 4.02 (t, *J* = 8.3 Hz, 1H, H-3), 3.90 (dd, *J* = 10.3, 9.4 Hz, 1H, H-3'), 3.87 – 3.80 (m, 2H, Et-OCH_2), 3.75 (dt, *J* = 10.4, 3.3 Hz, 1H, H-5'), 3.34 (dd, *J* = 10.3, 3.7 Hz, 1H, H-2'), 1.30 (td, *J* = 7.1, 1.1 Hz, 3H, Et-CH_3), 0.97 (td, *J* = 7.1, 1.1 Hz, 3H, Et-CH_3). **^{13}C{^{1}H} NMR** (101 MHz, $CDCl_3$): δ 168.1 (C=O), 167.3 (C=O), 165.0 (C=O), 154.4 (C=O), 143.3 (C), 143.1 (C), 141.47 (C), 141.45 (C), 137.2 (C), 137.1 (C), 133.8 (CH), 130.0 (CH), 129.2 (C), 128.8 (CH), 128.54 (CH), 128.53 (2 x CH), 128.13 (CH), 128.10 (CH), 128.1 (CH), 127.9 (CH), 127.4 (2 x CH), 125.2 (CH), 125.0 (CH), 120.3 (CH), 120.2 (CH), 97.6 (C-1'), 96.4 (d, *J* = 4.9 Hz, C-1), 81.9 (C-3), 77.3 (C-3'), 75.4 (Ph*C*H_2), 74.94 (C-4), 74.86 (Ph*C*H_2), 74.5 (C-5), 74.4 (C-4'), 73.2 (d, *J* = 8.4 Hz, C-2), 70.5 (Fmoc-*C*H_2), 68.6 (C-5'), 64.7 (d, *J* = 6.2 Hz, Et-O*C*H_2), 64.5 (d, *J* = 6.0 Hz, Et-O*C*H_2), 63.2 (C-6'), 62.8 (C-2'), 53.1 (OCH_3), 46.8

(Fmoc-*C*H), 41.0 (AcCl-*C*H_2), 16.0 (d, *J* = 7.3 Hz, Et-*C*H_3), 15.7 (d, *J* = 7.0 Hz, Et-*C*H_3). **^{31}P NMR** (162 MHz, $CDCl_3$): δ -2.80 (s, 1P, α), -3.29 (q, *J* = 7.7 Hz, 1P, β). **ESI-HRMS** for $C_{55}H_{61}O_{18}N_4ClP$ $(M+NH_4)^+$ calculated: 1131.3402; found: 1131.3414.

3. Automated Synthesis

3.1 Materials and Measurements

Solvents for dissolving all building blocks and making of solutions were taken from Solvent Dispensing System (J.C. Meyer). Wash solvents were HPLC grade. Prior to automated synthesis, the building blocks were co-evaporated three times with toluene and dried for at least 1 hour under high vacuum. All solutions were freshly prepared and kept under argon during the automation process. Isolated yields of products were calculated on the basis of resin loading. Functionalized resin **2** was synthesized and resin loading (0.45 mmol/g) was determined as previously reported.[3]

3.2 Preparation of Stock Solutions

Building Block Solution: Glycosyl phosphate building block (0.0653 mmol, 4.5 eq per cycle) was dissolved in anhydrous CH_2Cl_2 (1 mL per cycle).

Acidic Wash/Activator Solution: TMSOTf (0.9 mL, 5.0 mmol) was added to 40 mL of anhydrous CH_2Cl_2.

Pre-capping Solution: Pyridine (10 mL) was added to 90 mL of DMF.

Capping Solution: Methanesulfonic acid (1.2 mL, 18.5 mmol) and acetic anhydride (6 mL, 63.5 mmol) were added to 50 mL of anhydrous CH_2Cl_2.

Lev Deprotection Solution: $N_2H_4 \cdot AcOH$ (725 mg, 7.87 mmol) was dissolved in 50 mL of a 4:1:0.25 mixture of pyridine/acetic acid/water.

Fmoc Deprotection Solution: Et_3N (2.5 mL) was added to 47.5 mL anhydrous DMF.

AcCl Deprotection Solution: Thiourea (2.5 g, 32.84 mmol) was dissolved in 55 mL of a 10:1 mixture of 2-methoxyethanol/pyridine.

Sulfation Solution: $SO_3 \cdot TMA$ (900 mg, 6.5 mmol) was added to 16 mL of DMF and sonicated until dissolved.

3.3 Modules for Automated Synthesis

Initiation:

The resin loaded in the reaction vessel was washed with DMF, THF, and CH_2Cl_2 (3 x 2 mL for 25 s, respectively). The resin was then swollen in CH_2Cl_2 (2 mL) for 20 minutes while the temperature of the reaction vessel was cooled to -20 °C.

Module A: Acidic Washing

Once the temperature of the reaction vessel was adjusted -20 °C, **Acidic Wash Solution** (1 mL) was delivered to the reaction vessel. After bubbling for 3 minutes, the solution was drained. Finally, the resin was washed with CH_2Cl_2 (25 mL) for 25 s and drained.

Module B: Glycosylation for glycosyl phosphate

Upon draining the CH_2Cl_2 in the reaction vessel, the **Building Block Solution** (1 mL) was delivered to the reaction vessel. After the temperature reached the desired temperature (T_1), **Activator Solution** (1 mL) was delivered dropwise to the reaction vessel. The glycosylation mixture was incubated for the selected duration (t_1) at the desired T_1, then the reaction temperature was linearly ramped to T_2. Once T_2 was reached, the reaction mixture was incubated for an additional time (t_2). Once the incubation time was finished, the reaction mixture was drained to fraction collector and the resin was washed with CH_2Cl_2 (6 x 2 mL for 25 s).

Module C: Capping

The resin was washed with DMF (2 x 2 mL for 25 s) and the temperature of the reaction vessel was adjusted to 30 °C. **Pre-capping Solution** (2 mL) was delivered to the reaction vessel and drained after 1 minute. The resin was then washed with CH_2Cl_2 (3 x 2 mL for 25 s). Upon washing, **Capping Solution** (2 mL) was delivered and the resin and the reagents are incubated for 10 minutes. The solution was drained from the reaction vessel and the pre-capping and capping steps were repeated once more. Finally, the solution was drained and the resin was washed with CH_2Cl_2 (3 x 2 mL for 25 s).

Module D: Fmoc Deprotection

The resin was washed with DMF (3 x 2 mL for 25 s) and the temperature of the reaction vessel was adjusted to 25 °C. **Fmoc Deprotection Solution** (2 mL) was delivered to the reaction vessel and the reaction was incubated for 5 minutes. The reaction solution was drained and the resin was washed with DMF (3 x 2 mL for 25s). Then, fresh **Fmoc Deprotection Solution** (2 mL) was delivered and the process was repeated twice. Finally, the resin was washed with DMF (3 x 2 mL for 25 s) and CH_2Cl_2 (3 x 2 mL for 25 s). The temperature of the reaction vessel was decreased to -20 °C for the next module.

Module E: Lev Deprotection

The resin was washed with CH_2Cl_2 (3 x 2 mL for 25 s) and the temperature of the reaction vessel was adjusted to 40 °C. **Lev Deprotection Solution** (2 mL) was delivered to the reaction vessel and the reaction was incubated for five minutes. The reaction solution was drained from the reactor vessel and the resin was washed with CH_2Cl_2 (3 x 2 mL for 25 s). Then, fresh **Lev Deprotection Solution** (2 mL) was delivered and the process was repeated twice. Finally, the resin was washed with DMF, THF, and CH_2Cl_2 (3 x 2 mL for 25 s, respectively).

Module F: AcCl Deprotection

The resin was first washed with CH_2Cl_2 (3 x 2 mL for 25 s) then **ClAc Deprotection Solution** (2mL) was delivered to the reaction vessel. The temperature of the reaction vessel was then adjusted to and maintained at 80 °C. After 22 min, the reaction solution was drained from the reactor vessel. The resin was washed with DMF (3 x 2 mL for 15 s). Then, fresh **ClAc Deprotection Solution** (2 mL) was delivered and the process was repeated once more. Finally, the resin was washed with DMF (3 x 2 mL for 25 s) and CH_2Cl_2 (5 x 2 mL for 25 s).

Module G: Sulfation

The resin was first washed with CH_2Cl_2 (3 x 2 mL for 15 s) then **Sulfation Solution** (2 mL) was delivered to the reaction vessel. The temperature of the reaction vessel was then adjusted to and maintained at 90 °C. After 20 minutes the reaction solution was drained from the reaction vessel. Then, fresh **Sulfation Solution** (2 mL) was added and the process was repeated once more. Upon completion, the resin was washed with DMF (3 x 2 mL for 25 s).

Module H: Cleavage from Solid Support

The resin was transferred into a 5 mL disposable syringe (with a frit) containing a small stir-bar. CH_2Cl_2 (3 mL) was taken up in the syringe and the mixture was stirred under light irradiation (PR160L-370nm lamp placed 5 cm from the syringe) for 14 h. The reaction solution was filtered and the solid was washed with CH_2Cl_2 (3 x 3 mL). The resulting solution was concentrated *in vacuo* to give the crude product.

3.4 *N*-Benzyloxycarbonyl-5-aminopentyl methyl (2-azido-3-*O*-benzyl-6-*O*-chloroacetyl-2-deoxy-4-*O*-fluorenylmethoxycarbonyl-α-D-glucopyranosyl)-(1→4)-(2-*O*-benzoyl-3-*O*-benzyl-β-D-glucopyranosyluronate)-(1→4)-(2-azido-3-*O*-benzyl-6-*O*-chloroacetyl-2-deoxy-D-glucopyranosyl)-(1→4)-(2-*O*-benzoyl-3-*O*-benzyl-β-D-glucopyranosyluronate) 9

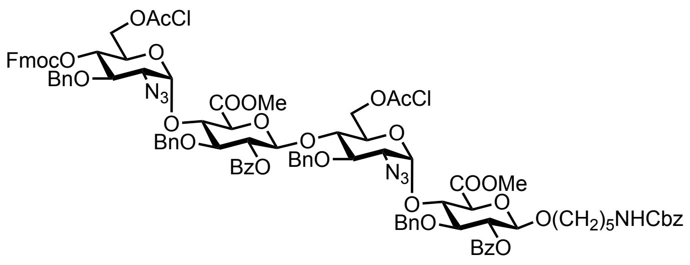

Resin **7** (32 mg, 0.0145 mmol, 1 eq) and phosphate donor **6** (73 mg, 0.0653 mmol, 4.5 eq per cycle) were used. After cleavage from solid support the crude product was analysed using normal phase analytical HPLC (YMC-diol-300-NP; Linear gradient: Hexane/EtOAc; 20-100% in 30 minutes) and purified using preparative HPLC (YMC-diol-300-NP; Linear gradient: Hexane/EtOAc; 20-100% in 30 minutes) to obtain protected tetrasaccharide **9** in 18% overall yield (5 mg).

Step	Module	Notes
1	**A**: Acidic wash	
2	**B**: Glycosylation x 2	T_1 = -15 °C; t_1 = 35 min
		T_2 = 0 °C; t_1 = 35 min
3	**C**: Capping	
4	**D**: Fmoc deprotection	
5	**A**: Acidic wash	
6	**B**: Glycosylation x 2	T_1 = -15 °C; t_1 = 35 min
		T_2 = 0 °C; t_1 = 35 min
7	**H**: Cleavage from solid support	

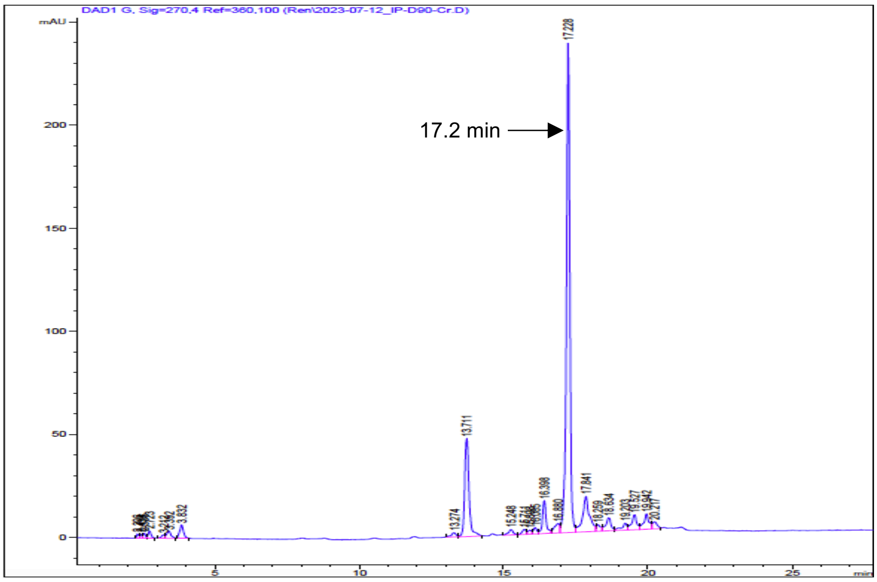

Figure 1. Analytical NP-HPLC trace (270 nm) of crude tetrasaccharide 9 (t_r = 17.2 min)

^{1}H NMR (600 MHz, $CDCl_3$): δ 8.12 – 8.08 (m, 2H, ArH), 8.02 – 8.00 (m, 2H, ArH), 7.76 (dd, *J* = 7.6, 4.1 Hz, 2H, ArH), 7.63 – 7.57 (m, 2H, ArH), 7.56 – 7.53 (m, 2H, ArH), 7.52 – 7.47 (m, 2H, ArH), 7.45 – 7.33 (m, 14H, ArH), 7.33 – 7.26 (m, 5H, ArH), 7.25 – 7.04 (m, 17H, ArH), 5.47 (d, *J* = 3.8 Hz, 1H, H-1'''), 5.46 (d, *J* = 3.9 Hz, 1H, H-1'), 5.41 (dd, *J* = 8.9, 7.8 Hz, 1H, H-2''), 5.26 (dd, *J* = 8.8, 7.2 Hz, 1H, H-2), 5.14 (d, *J* = 10.8 Hz, 1H, C*H*HPh), 5.07 (s, 2H, 2 x C*H*HPh), 4.81 (dd, *J* = 10.3, 9.1 Hz, 1H, H-4'''), 4.78 (d, *J* = 10.4 Hz, 1H, C*H*HPh), 4.75 (d, *J* = 10.9 Hz, 1H, C*H*HPh), 4.72 – 4.62 (m, 6H, H-1'', 5x C*H*HPh), 4.61 – 4.57 (m, 1H, NH), 4.55 (d, *J* = 7.2 Hz, 1H, H-1), 4.50 (dd, *J* = 10.6, 6.6 Hz, 1H, Fmoc-C*H*H), 4.35 – 4.22 (m, 6H, Fmoc-C*H*H, H-6a', H-6a''', H-4'', H-6b', H-6b'''), 4.19 (t, *J* = 6.9 Hz, 1H, Fmoc-CH), 4.17 – 4.14 (m, 1H, H-4), 4.13 – 4.08 (m, 3H, 3 x AcCl-C*H*H), 4.05 (d, *J* = 14.4 Hz, 1H, AcCl-C*H*H), 3.99 (d, *J* = 9.4 Hz, 1H, H-5''), 3.97 – 3.91 (m, 3H, H-3, H-3'', H-5), 3.90 (dd, *J* = 10.3, 9.1 Hz, 1H, H-3'''), 3.82 – 3.72 (m, 3H, Linker-OC*H*H, H-4', H-3'), 3.70 (dt, *J* = 10.5, 3.1 Hz, 1H, H-5'''), 3.54 (s, 3H, OCH_3), 3.43 – 3.33 (m, 2H, H-5', Linker-OC*H*H), 3.34 (dd, *J* = 10.3, 3.8 Hz, 1H, H-2'''), 3.23 – 3.18 (m, 1H, H-2'), 3.19 (s, 3H, OCH_3), 2.92 (q, *J* = 6.9 Hz, 1H, Linker-NCH_2), 1.51 – 1.40 (m, 2H, Linker-CH_2), 1.37 – 1.25 (m, 2H, Linker-CH_2), 1.22 – 1.11 (m, 2H, Linker-CH_2). **^{13}C{^{1}H} NMR** (101 MHz, $CDCl_3$): δ 168.6 (C=O), 167.9 (C=O), 167.4 (AcCl-C=O), 167.1 (AcCl-C=O), 165.1 (Bz-C=O), 164.8 (Bz-C=O), 156.4 (CBz-C=O), 154.3 (Fmoc-C=O), 143.30 (C), 143.1 (C), 141.5 (C), 141.4 (C), 138.1 (C), 137.3 (C), 137.13 (C), 137.11 (C), 136.8 (C), 134.1 (CH), 133.6 (CH), 130.0 (CH), 129.8 (CH), 129.6 (C), 129.1 (CH), 128.8 (C), 128.71 (CH), 128.66 (CH), 128.54 (CH), 128.51 (CH), 128.50 (CH), 128.45 (CH), 128.3 (CH), 128.23 (CH), 128.15 (CH), 128.12 (CH), 128.07 (CH), 127.98

(CH), 127.95 (CH), 127.91 (CH), 127.8 (CH), 127.7 (CH), 127.4 (CH), 125.2 (CH), 125.0 (CH), 120.3 (CH), 120.2 (CH), 101.3 (C-1, $^{1}J_{CH}$ = 165.0 Hz, from coupled HSQC), 101.2 (C-1'', $^{1}J_{CH}$ = 166.6 Hz, from coupled HSQC), 97.6 (C-1''', $^{1}J_{CH}$ = 180.0 Hz, from coupled HSQC), 97.4 (C-1', $^{1}J_{CH}$ = 180.0 Hz, from coupled HSQC), 82.7, 82.5 (C-3, C-3''), 77.82 (C-3' from HSQC), 77.75 (C-4' from HSQC), 77.4 (C-3''' from HSQC), 75.7 (Ph*C*H$_2$), 75.5, 75.4 (Ph*C*H$_2$), 75.2 (Ph*C*H$_2$), 74.7 (Ph*C*H$_2$), 74.60 (C-5''), 74.56 (C-4), 74.3 (C-4'''), 74.0 (C-5), 73.9 (C-2), 73.6 (C-2''), 70.5 (Fmoc-*C*H$_2$), 69.9 (Linker-O*C*H$_2$), 68.9 (C-5'), 68.4 (C-5''''), 66.7 (CBz-*C*H$_2$), 63.2, 63.1 (C-6', C-6'''), 62.73, 62.68 (C-2', C-2'''), 53.0 (OCH$_3$), 52.2 (OCH$_3$), 46.8 (Fmoc-*C*H), 41.0, 40.9 (2 x AcCl-*C*H$_2$, Linker-N*C*H$_2$), 29.5 (Linker-*C*H$_2$), 28.9 (Linker-*C*H$_2$), 23.1 (Linker-*C*H$_2$). **ESI-HRMS** for $C_{100}H_{101}O_{29}N_7Cl_2Na$ $(M+Na)^+$ calculated: 1957.5952; found: 1957.5968.

3.5 *N*-Benzyloxycarbonyl-5-aminopentyl methyl (2-azido-3-*O*-benzyl-2-deoxy-4-*O*-fluorenylmethoxycarbonyl-6-*O*-chloroacetyl-α-D-glucopyranosyl)-(1→4)-(2-*O*-benzoyl-3-*O*-benzyl-β-D-glucopyranosyluronate)-(1→4)-(2-azido-3-*O*-benzyl-6-*O*-chloroacetyl-2-deoxy-D-glucopyranosyl)-(1→4)-(2-*O*-benzoyl-3-*O*-benzyl-β-D-glucopyranosyluronate) 10

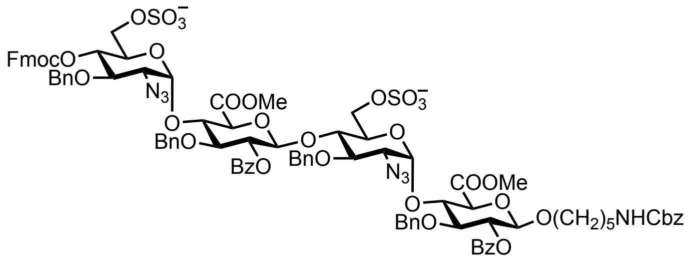

Resin **7** (32 mg, 0.0145 mmol, 1 eq) and phosphate donor **6** (73 mg, 0.0653 mmol, 4.5 eq per cycle) were used. After cleavage from solid support the crude product was analysed using reverse phase analytical HPLC (C18; Linear gradient: 0.01 M NH_4HCO_3 in water/MeCN; 5-100% in 30 minutes) and purified using manual reverse phase column (HyperSep™ C18 cartridge; Linear gradient: H_2O/MeCN; 20-60%) to obtain sulfated tetrasaccharide **10** in 3% overall yield (1.0 mg). *Note: Fractions were analysed by mass spectrometry (negative mode) for purity before combining. Compound degraded overnight in $CDCl_3$.*

^{1}H NMR (400 MHz, $CDCl_3$) selected signals: δ 3.56 (s, 3H, OCH$_3$), 3.02 (s, 3H, OCH$_3$), 2.73 (s, 9H, N(CH$_3$)$_3$), 2.72 (s, 9H, N(CH$_3$)$_3$). **ESI-HRMS** for $(C_{96}H_{97}N_7O_{33}S_2)^{2-}$ $(M)^{2-}$ calculated: 970.2801; found: 970.2797.

Step	Module	Notes
1	**A**: Acidic wash	
2	**B**: Glycosylation x 2	T_1 = -15 °C; t_1 = 35 min<br>T_2 = 0 °C; t_1 = 35 min
3	**C**: Capping	
4	**D**: Fmoc deprotection	
5	**A**: Acidic wash	
6	**B**: Glycosylation x 2	T_1 = -15 °C; t_1 = 35 min<br>T_2 = 0 °C; t_1 = 35 min
7	**C**: Capping	
8	**F**: AcCl deprotection	
9	**G**: Sulfation	
10	**H**: Cleavage from solid support	

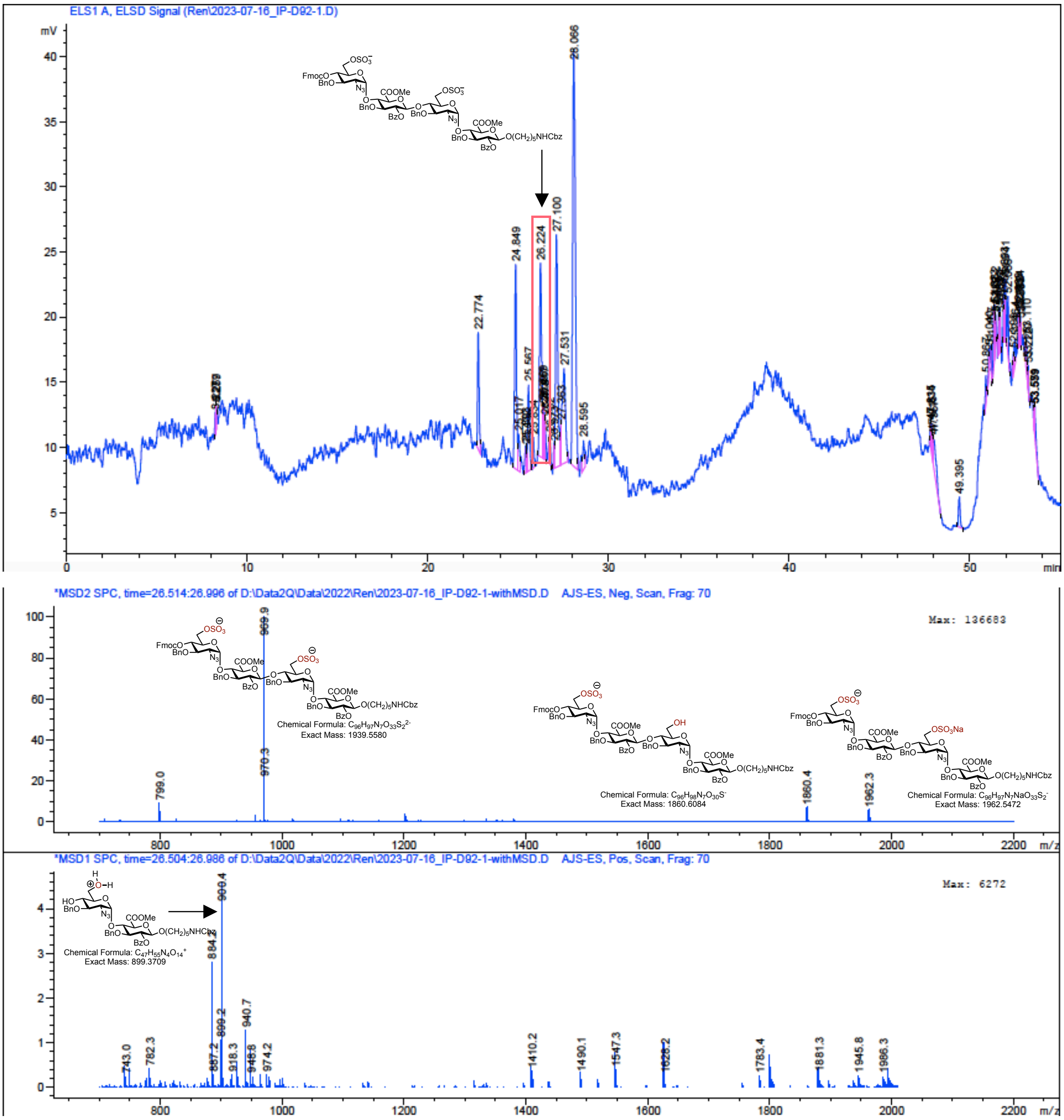

Figure 2. Analytical RP-HPLC trace of crude tetrasaccharide **10**. Top- ELSD trace (t_r = 26 min); Middle- MSD negative mode showing fully and partially sulfated tetrasaccharide for t_r = 26 min (26.514 to 26.996 min); Bottom- MSD positive mode for t_r = 26 min (time = 26.504 to 26.986 min).

4. Spectra

4.1 Compound 2 NMR

^{1}H NMR (400 MHz, Chloroform-*d*) 2

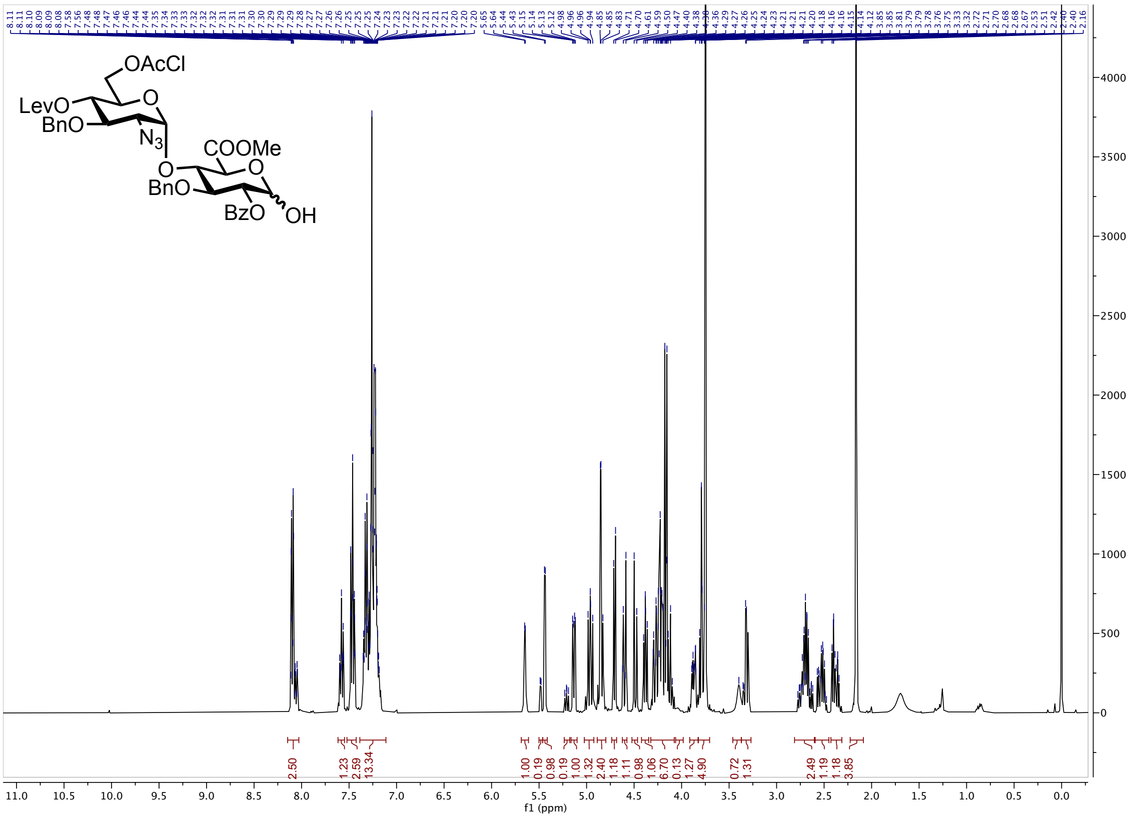

^{13}C{^{1}H} NMR (101 MHz, Chloroform-*d*) 2

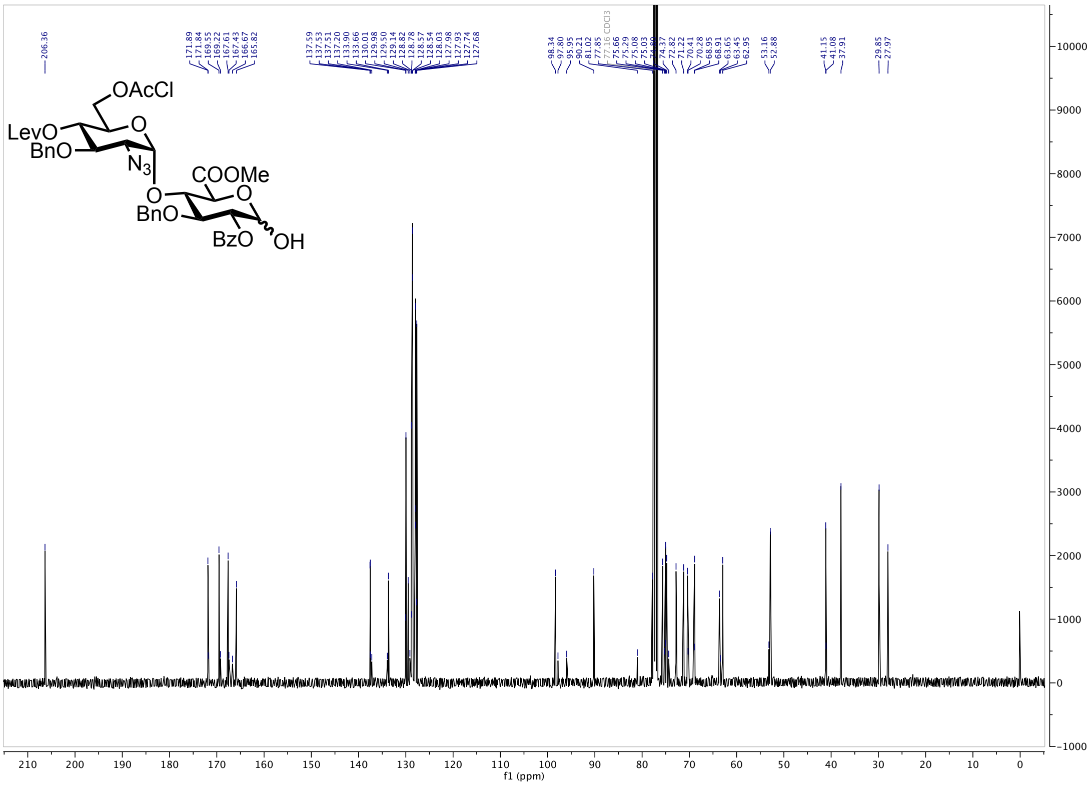

COSY NMR (400 MHz, Chloroform-*d*) 2

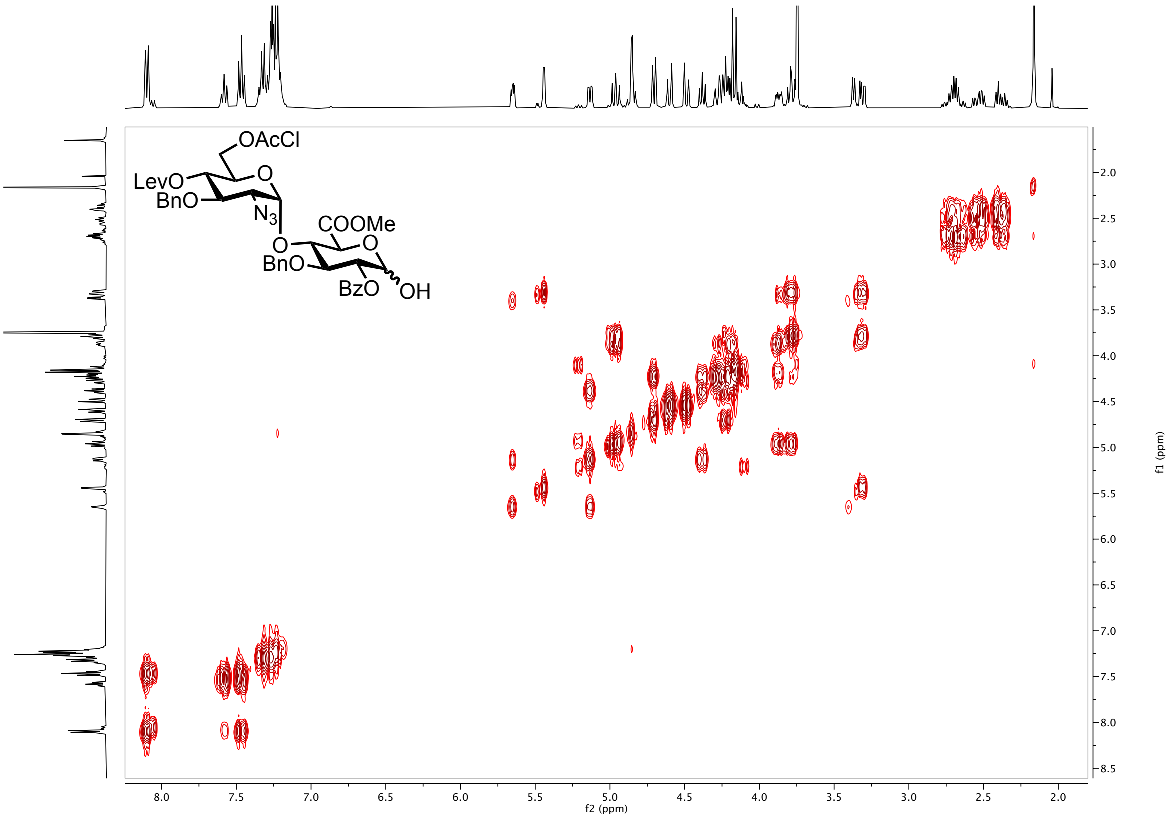

HSQC NMR (400 MHz x 101 MHz, Chloroform-*d*) 2

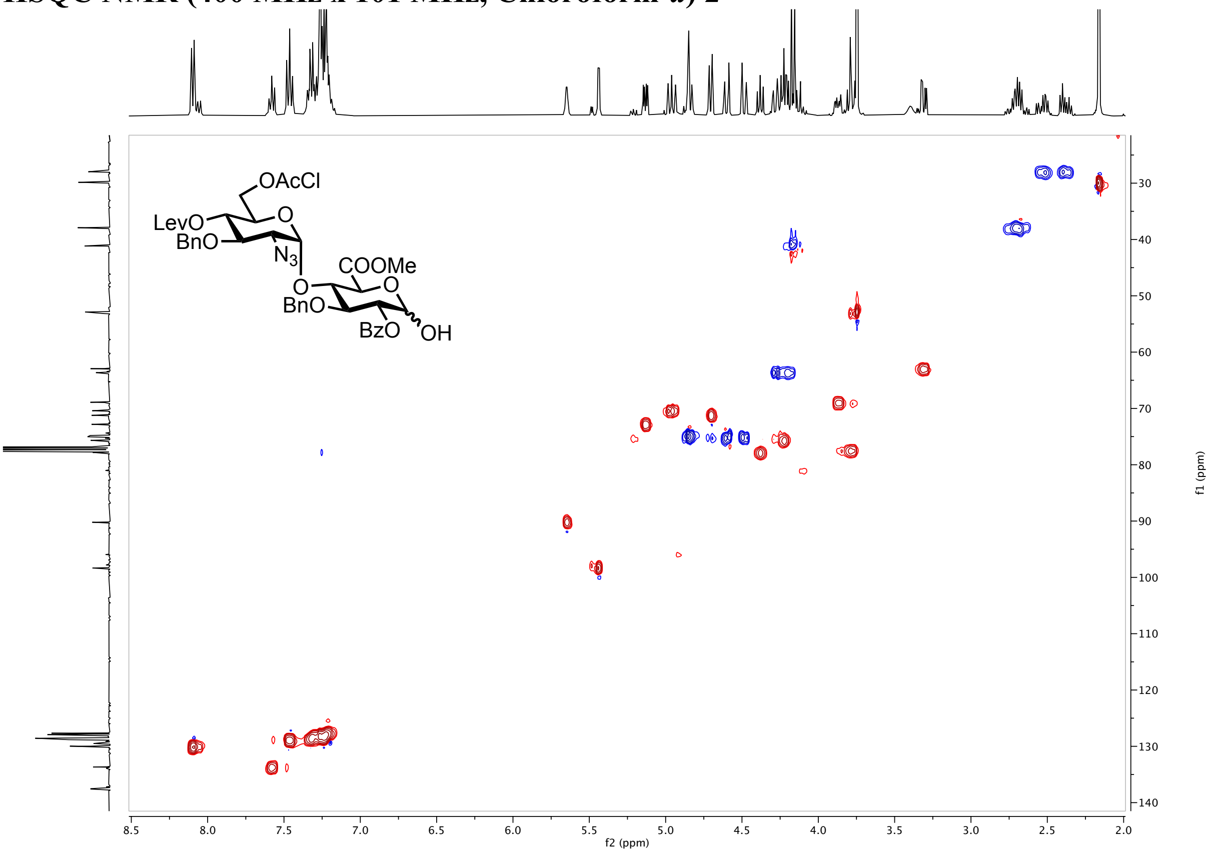

HMBC NMR (400 MHz x 101 MHz, Chloroform-*d*) 2

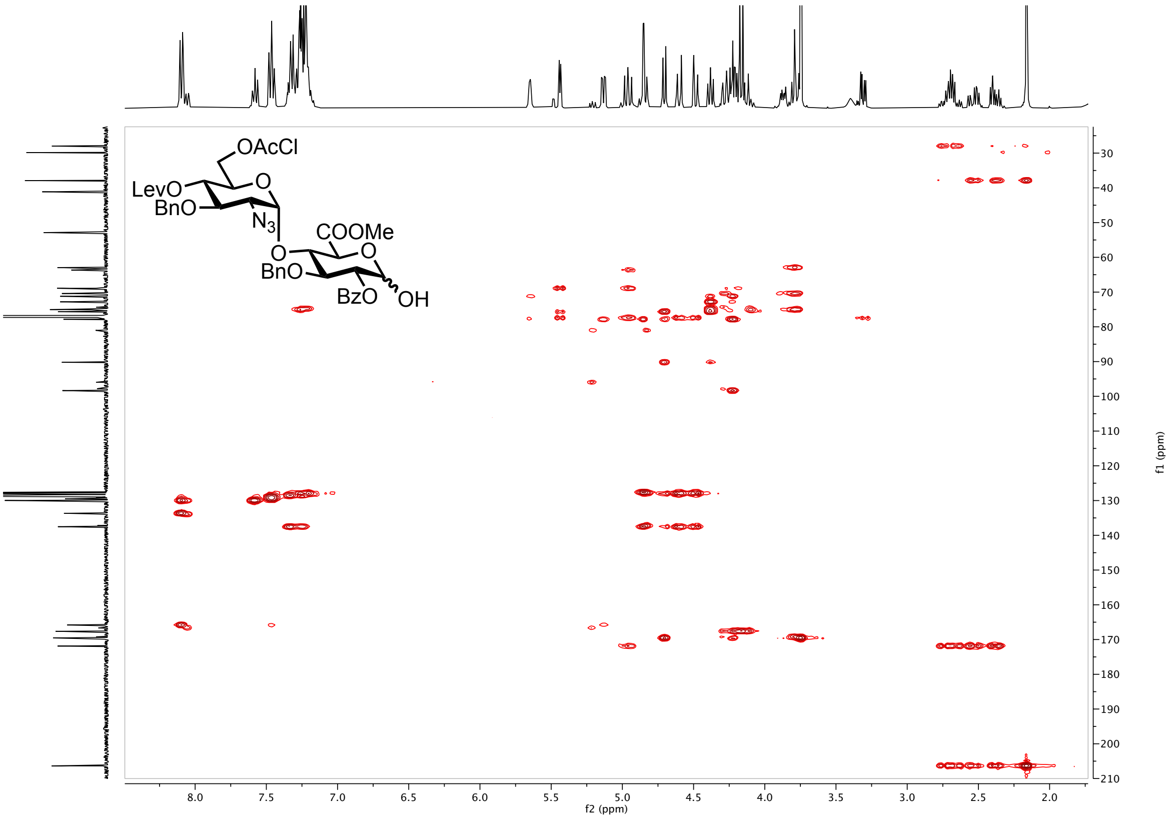

4.2 Compound 4 NMR

^{1}H NMR (400 MHz, Chloroform-*d*) 4

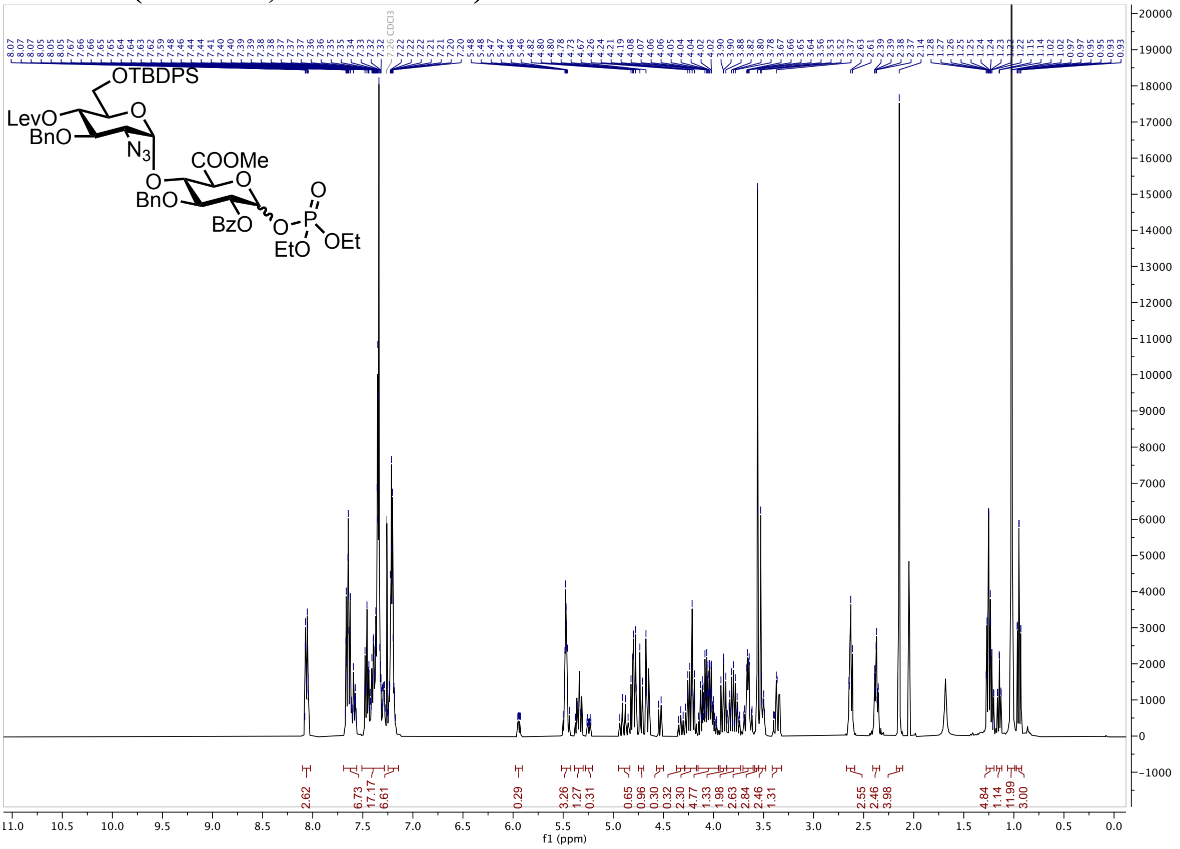

$^{31}P\{^{1}H\}$ NMR (162 MHz, Chloroform-*d*) 4

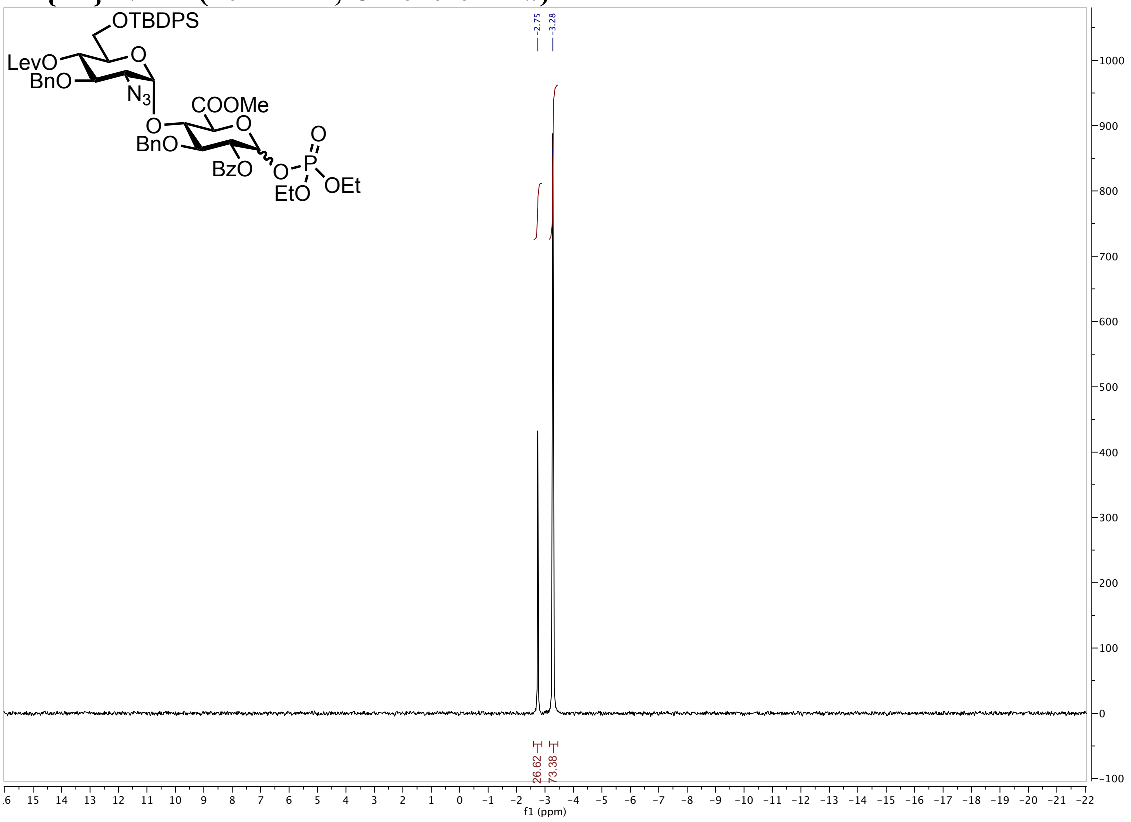

$^{13}C\{^{1}H\}$ NMR (101 MHz, Chloroform-*d*) 4

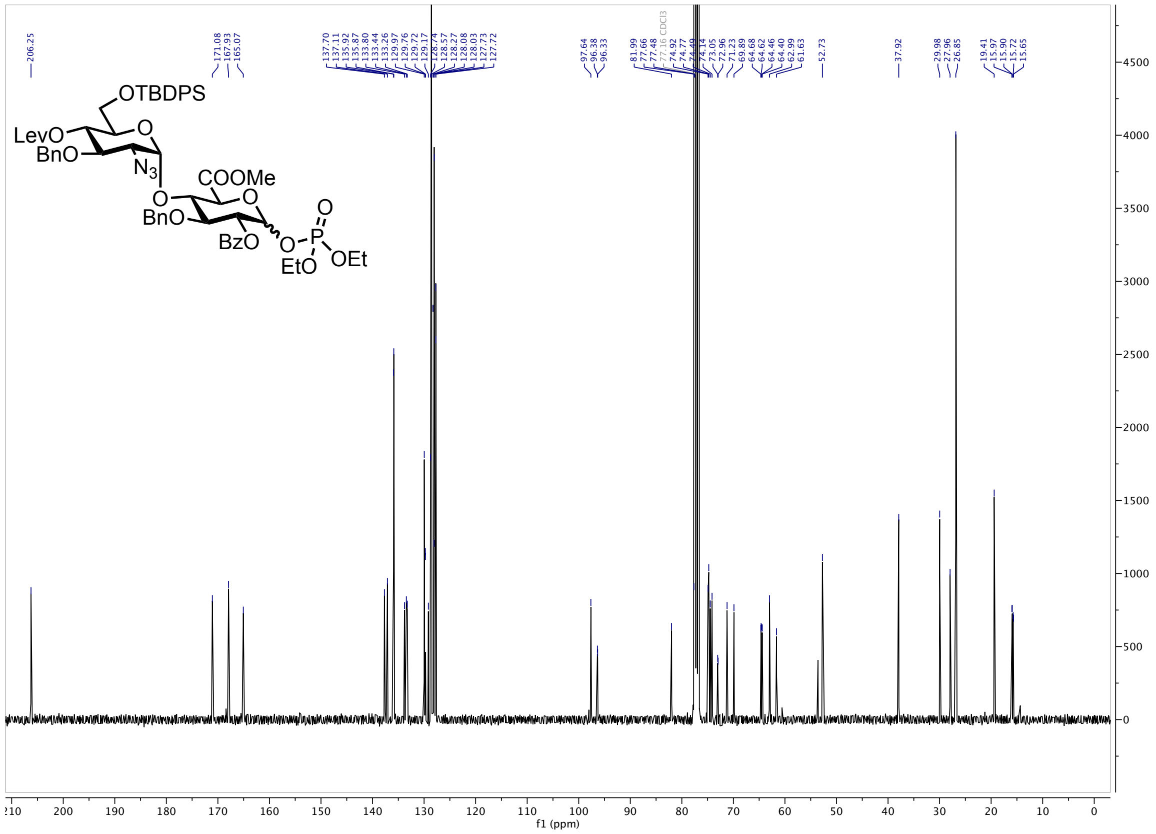

COSY NMR (400 MHz, Chloroform-*d*) 4

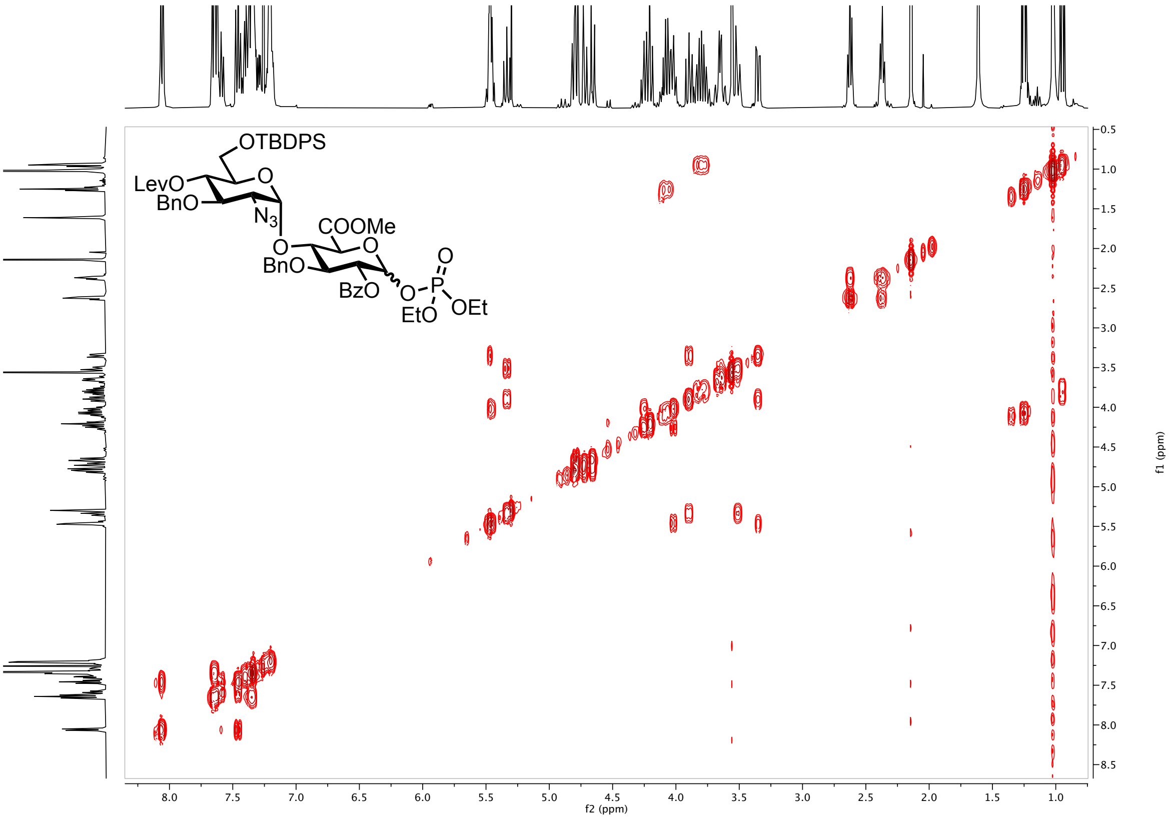

HSQC NMR (400 MHz x 101 MHz, Chloroform-*d*) 4

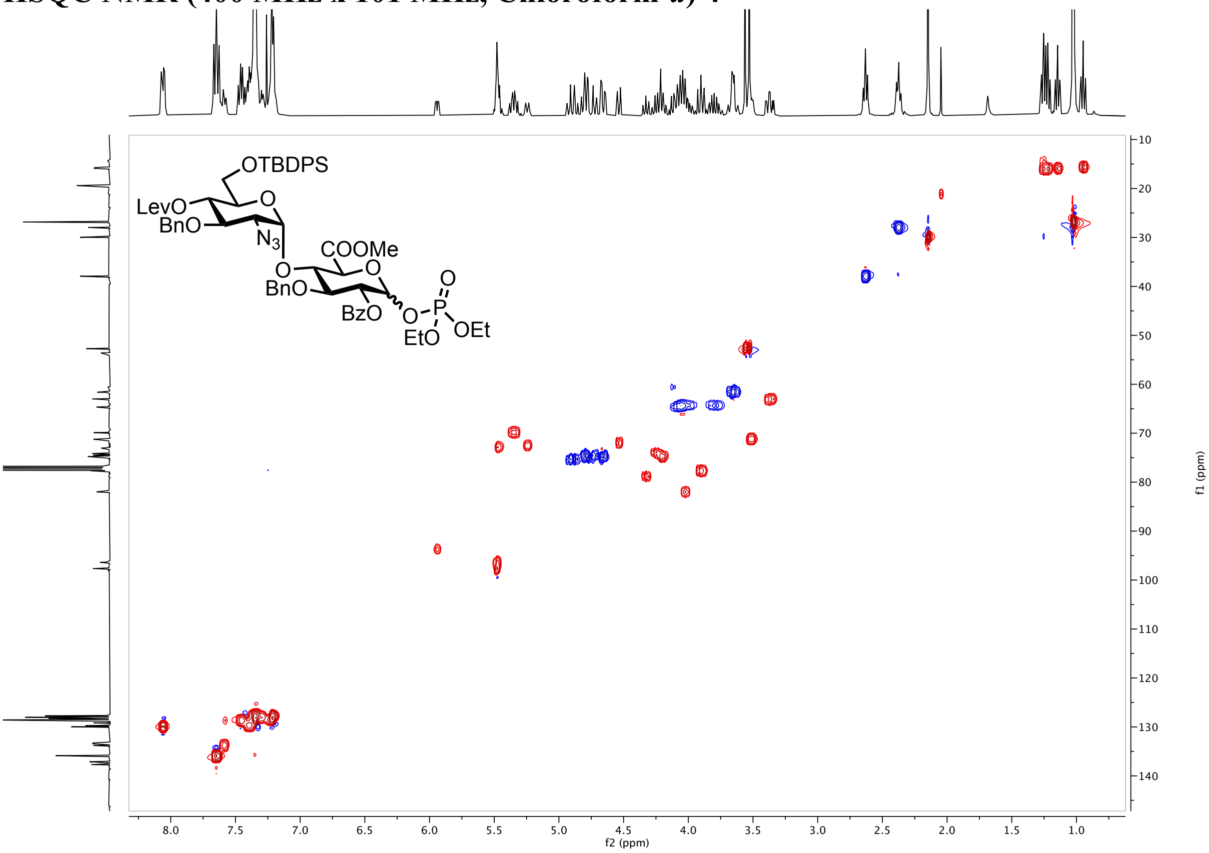

HMBC NMR (400 MHz x 101 MHz, Chloroform-*d*) 4

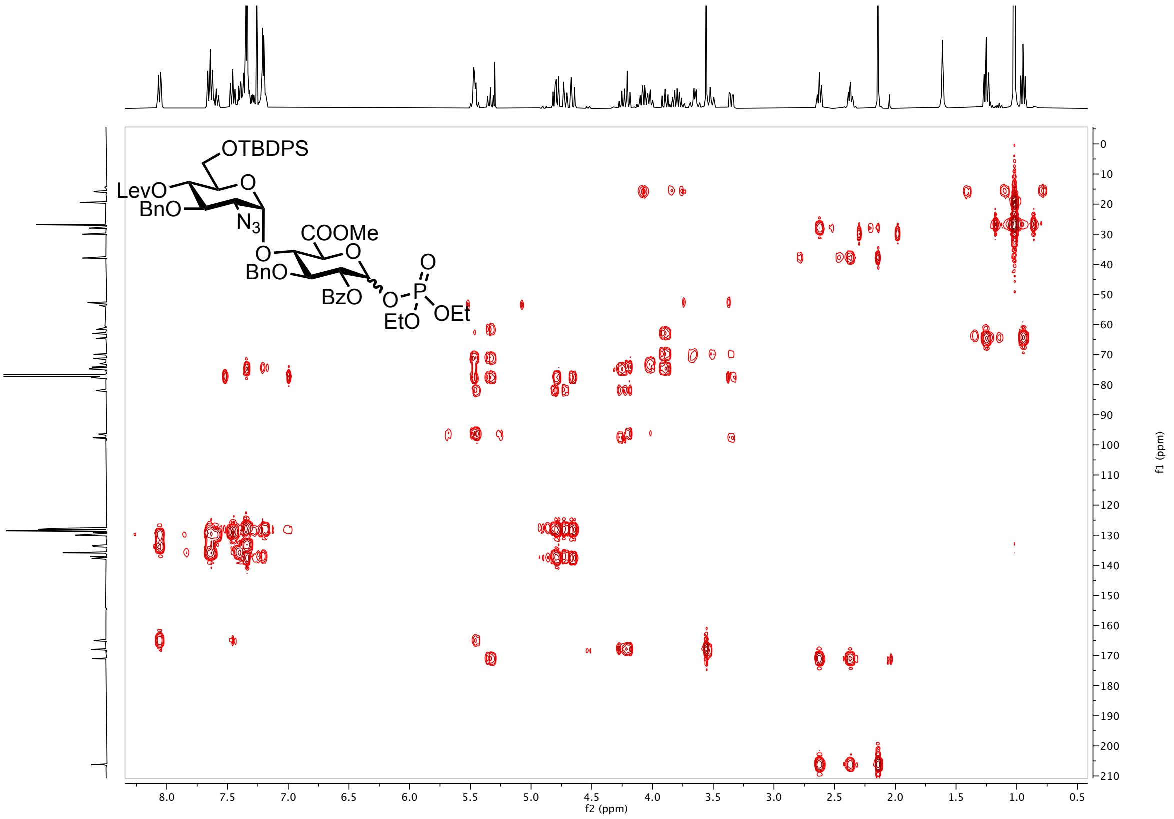

4.3 Compound 5 NMR

^{1}H NMR (400 MHz, Chloroform-*d*) 5

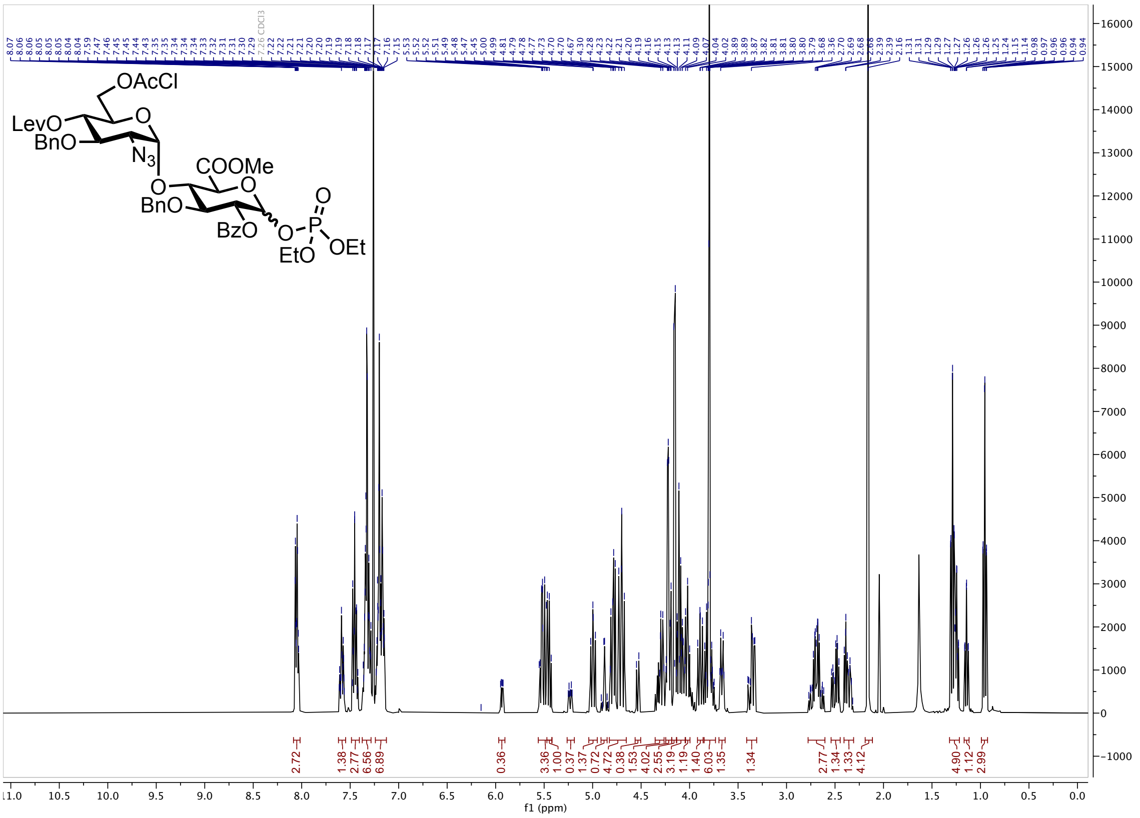

$^{31}P\{^1H\}$ NMR (162 MHz, Chloroform-*d*) 5

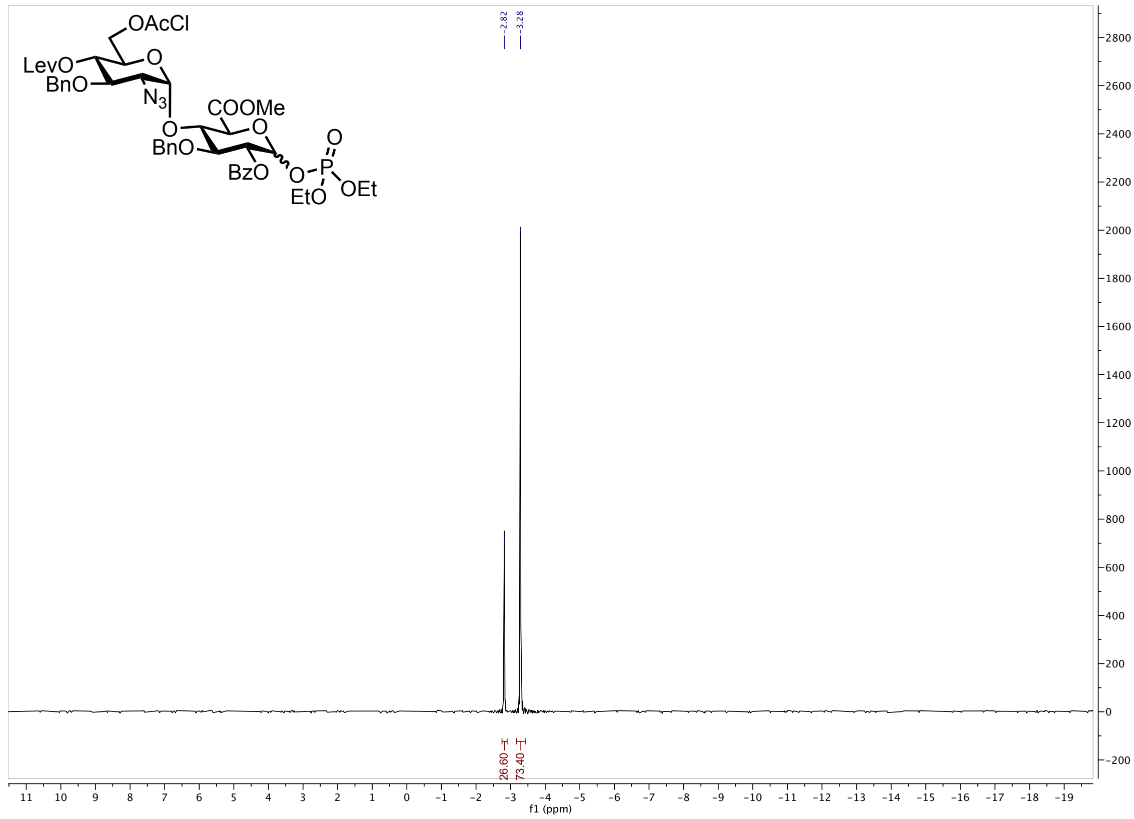

$^{13}C\{^1H\}$ NMR (101 MHz, Chloroform-*d*) 5

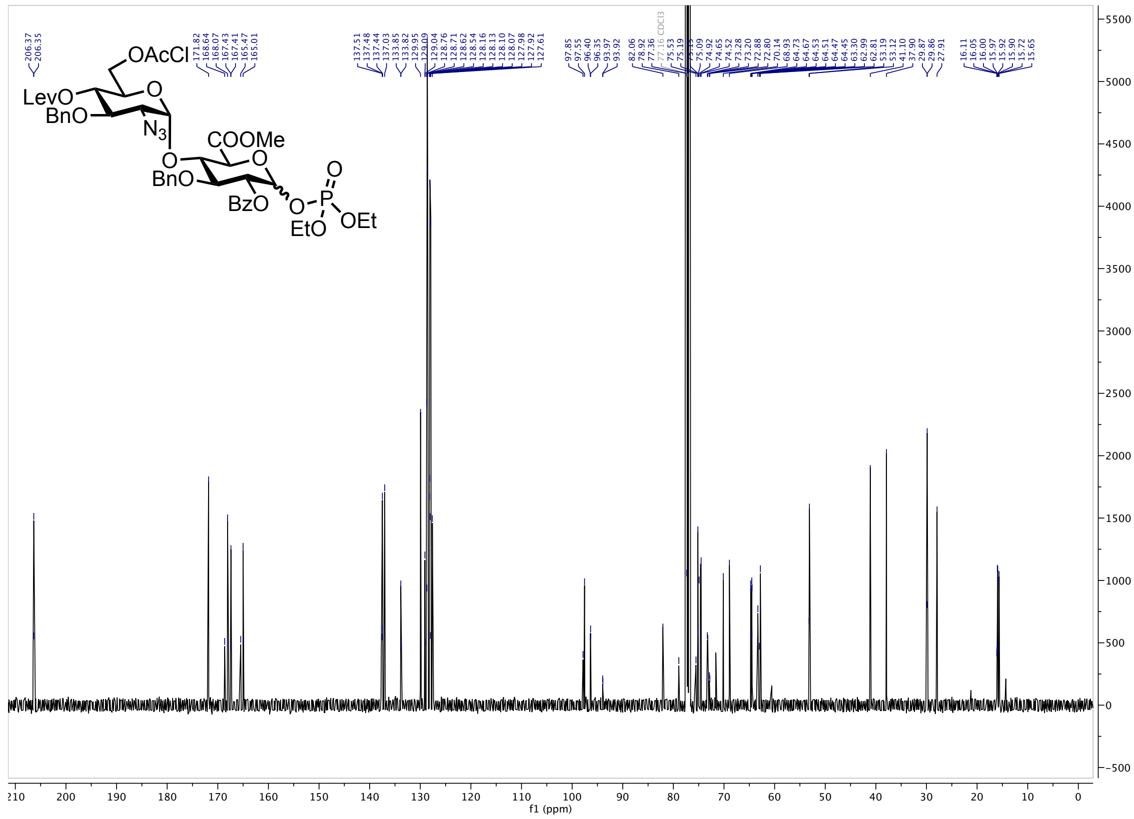

COSY NMR (400 MHz, Chloroform-*d*) 5

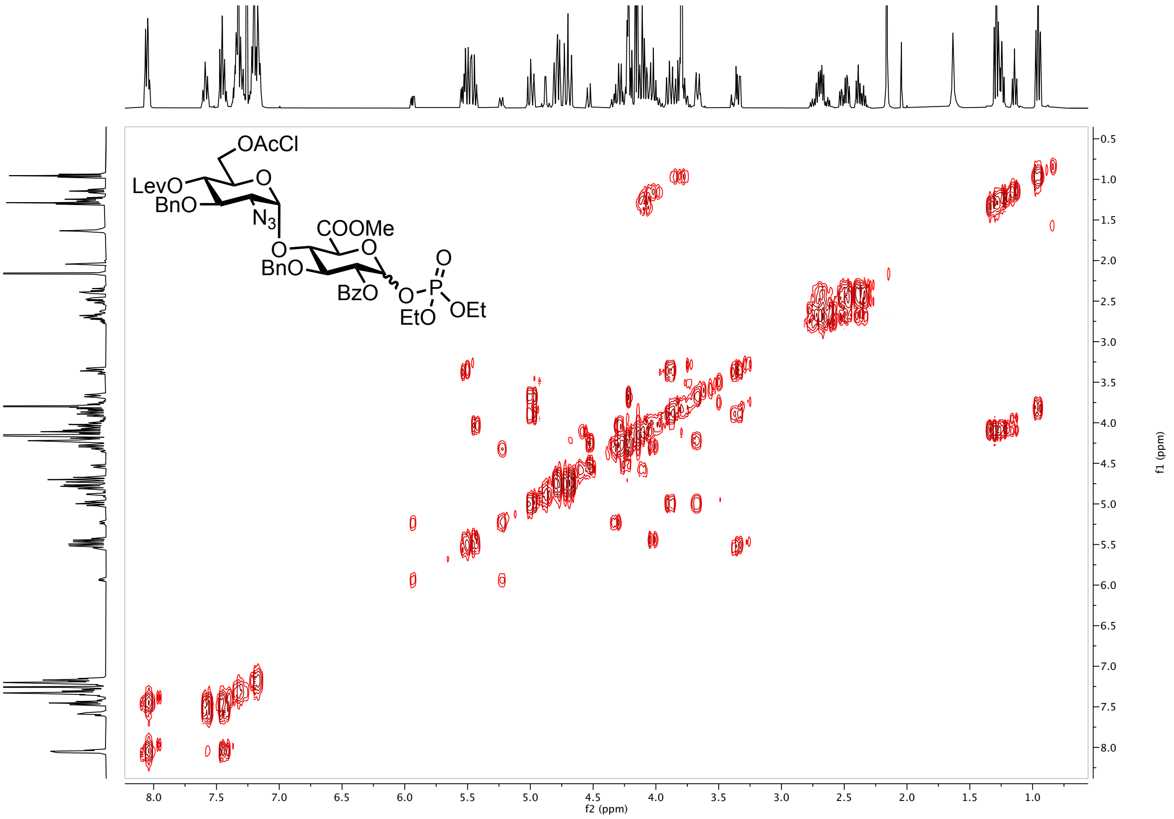

HSQC NMR (400 MHz x 101 MHz, Chloroform-*d*) 5

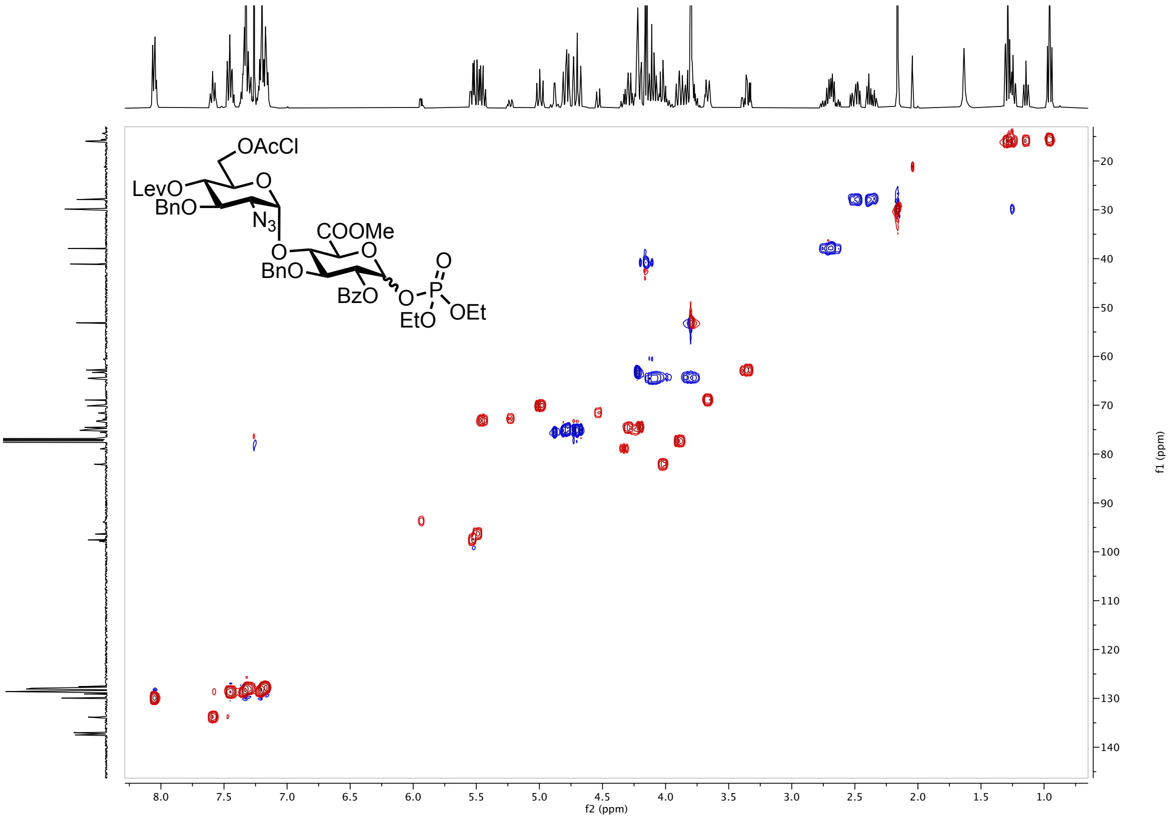

HMBC NMR (400 MHz x 101 MHz, Chloroform-*d*) 5

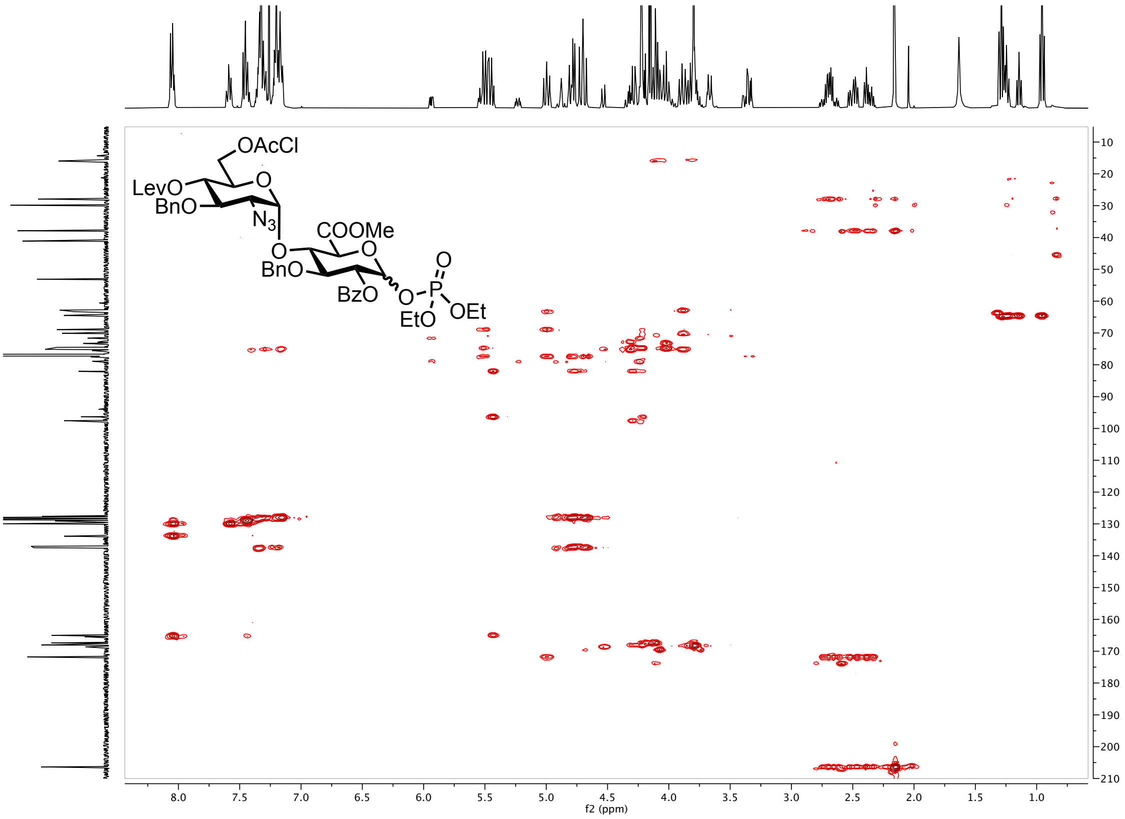

4.4 Compound 6 NMR

^{1}H NMR (400 MHz, Chloroform-*d*) 6

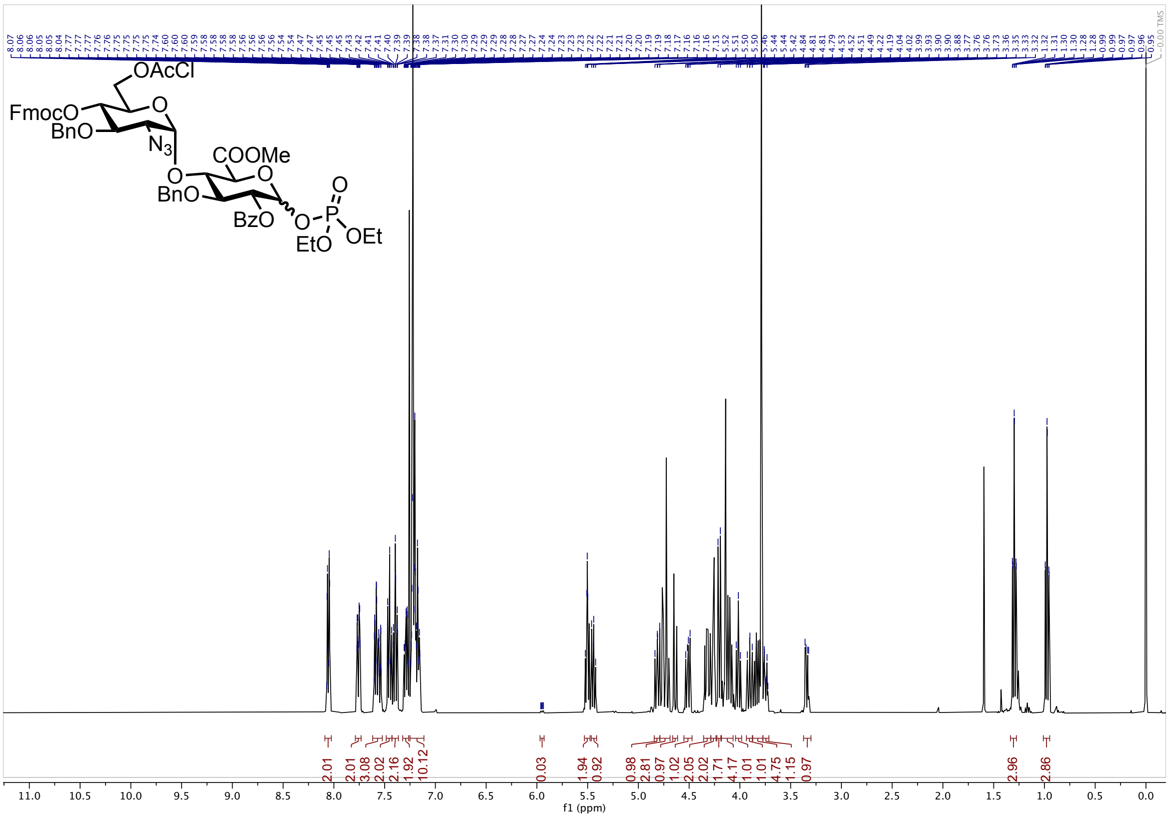

^{31}P NMR (162 MHz, Chloroform-*d*) 6

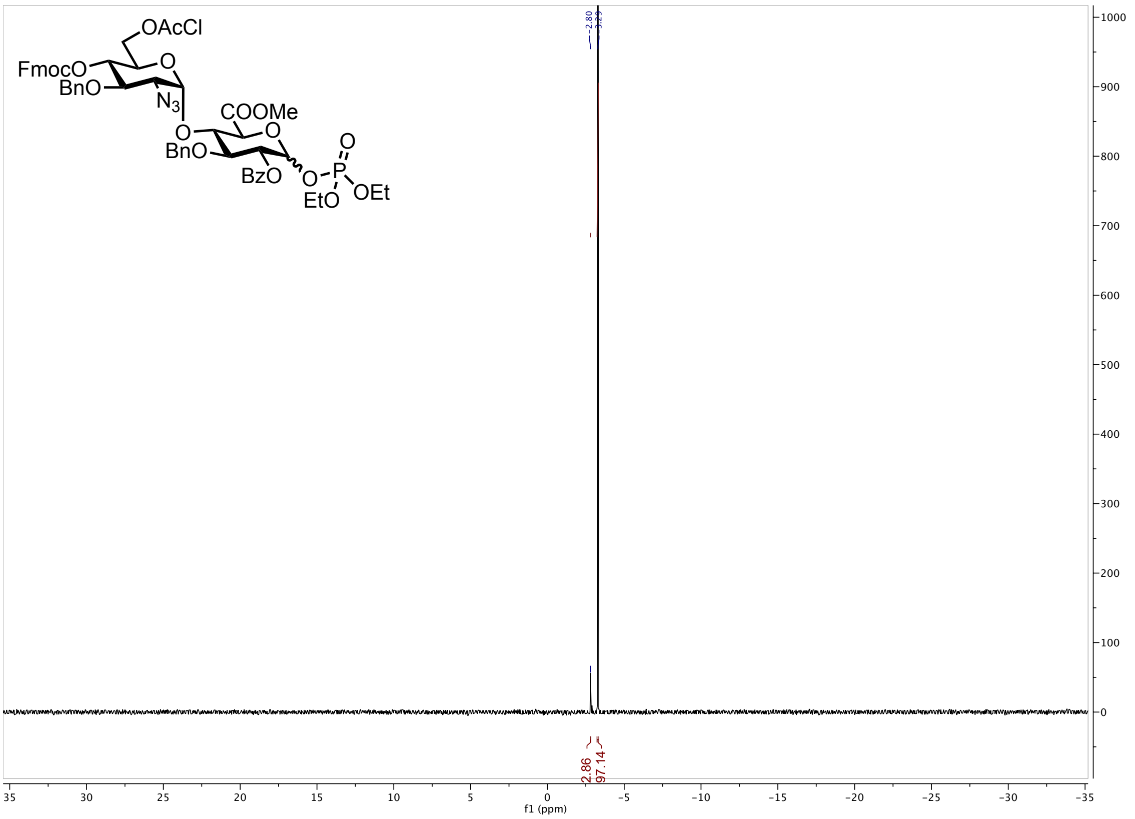

^{13}C{^{1}H} NMR (101 MHz, Chloroform-*d*) 6

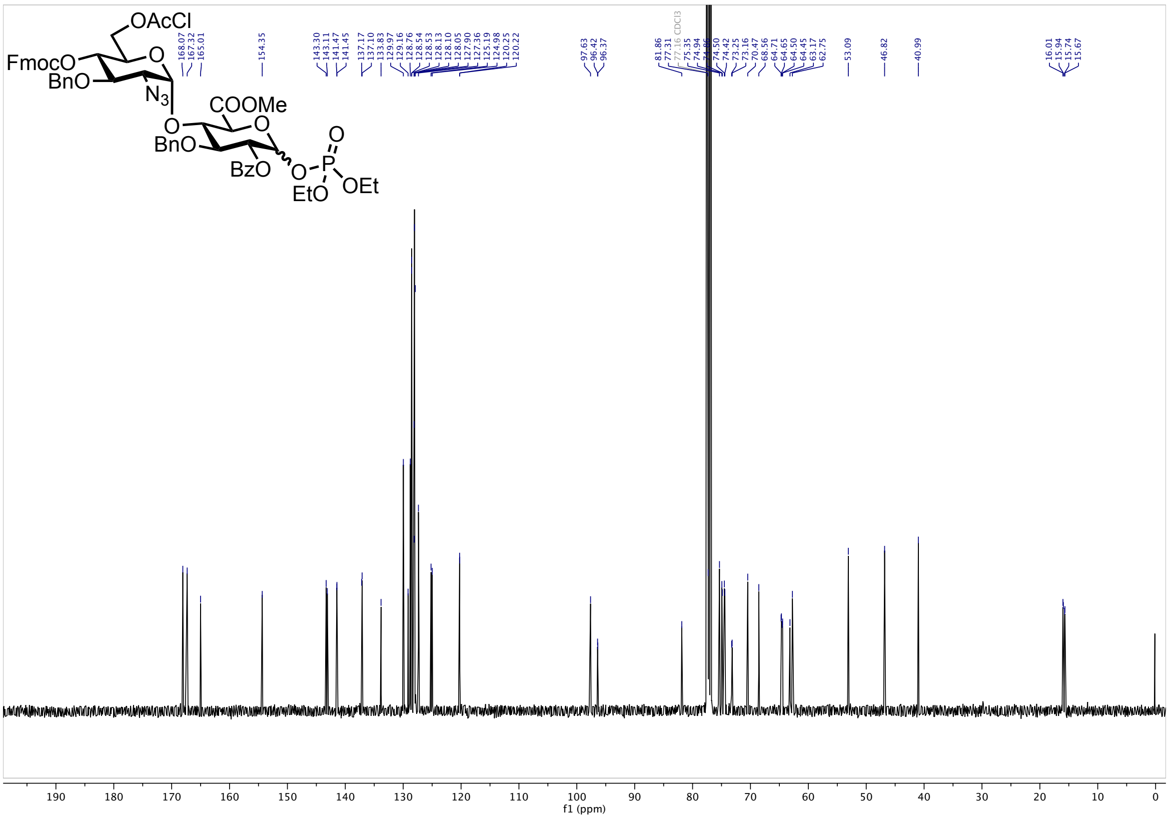

DEPT NMR (101 MHz, Chloroform-*d*) 6

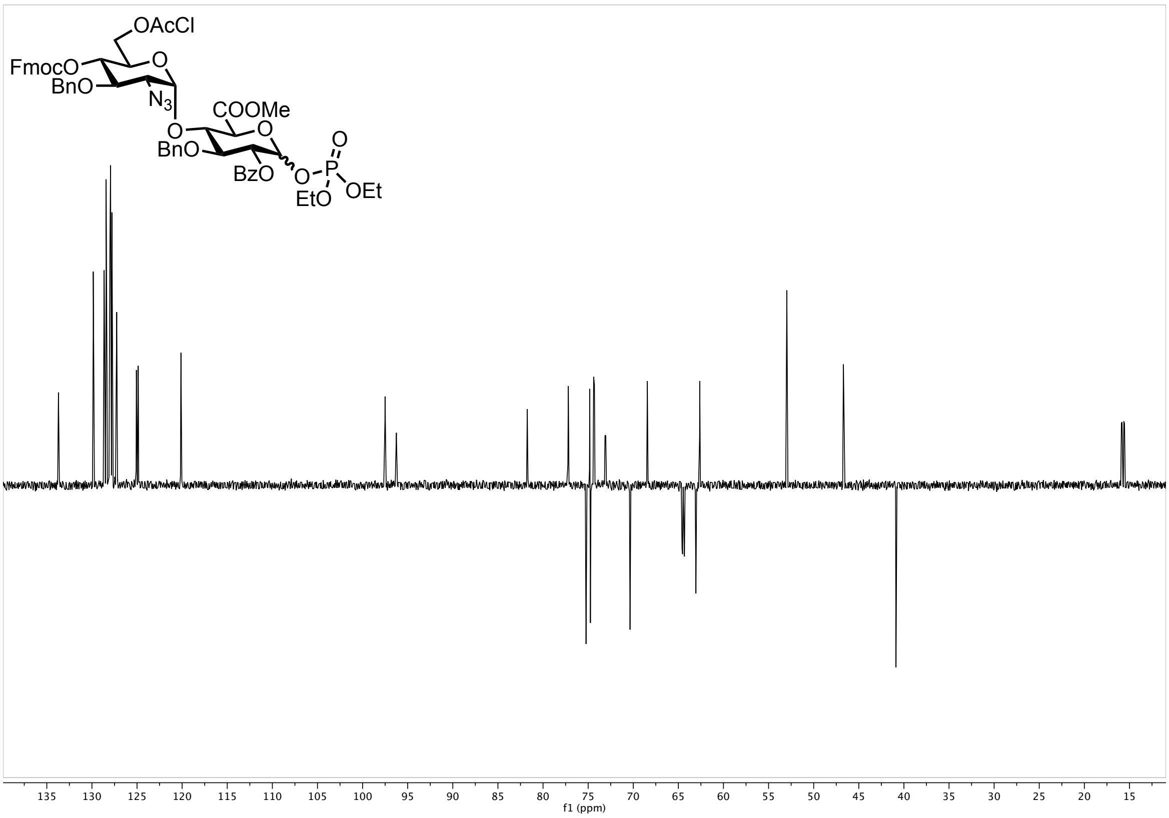

COSY NMR (400 MHz, Chloroform-*d*) 6

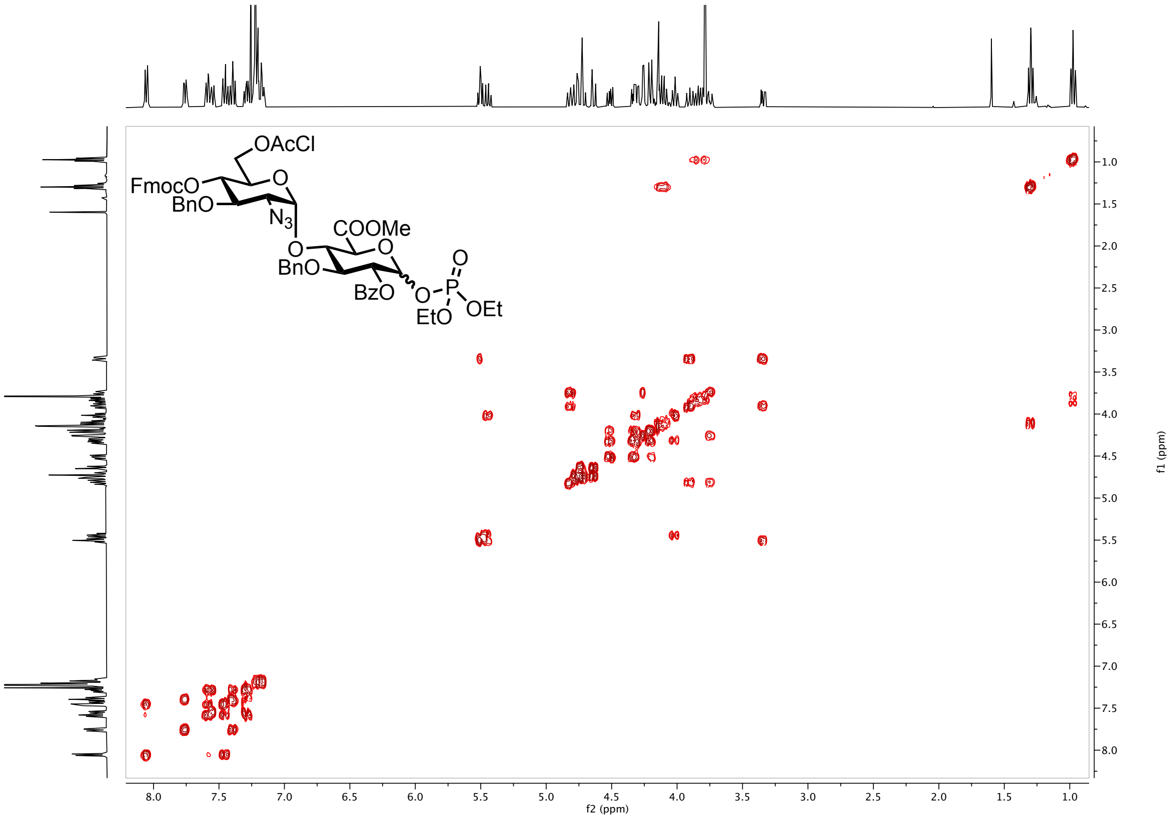

HSQC NMR (400 MHz x 101 MHz, Chloroform-*d*) 6

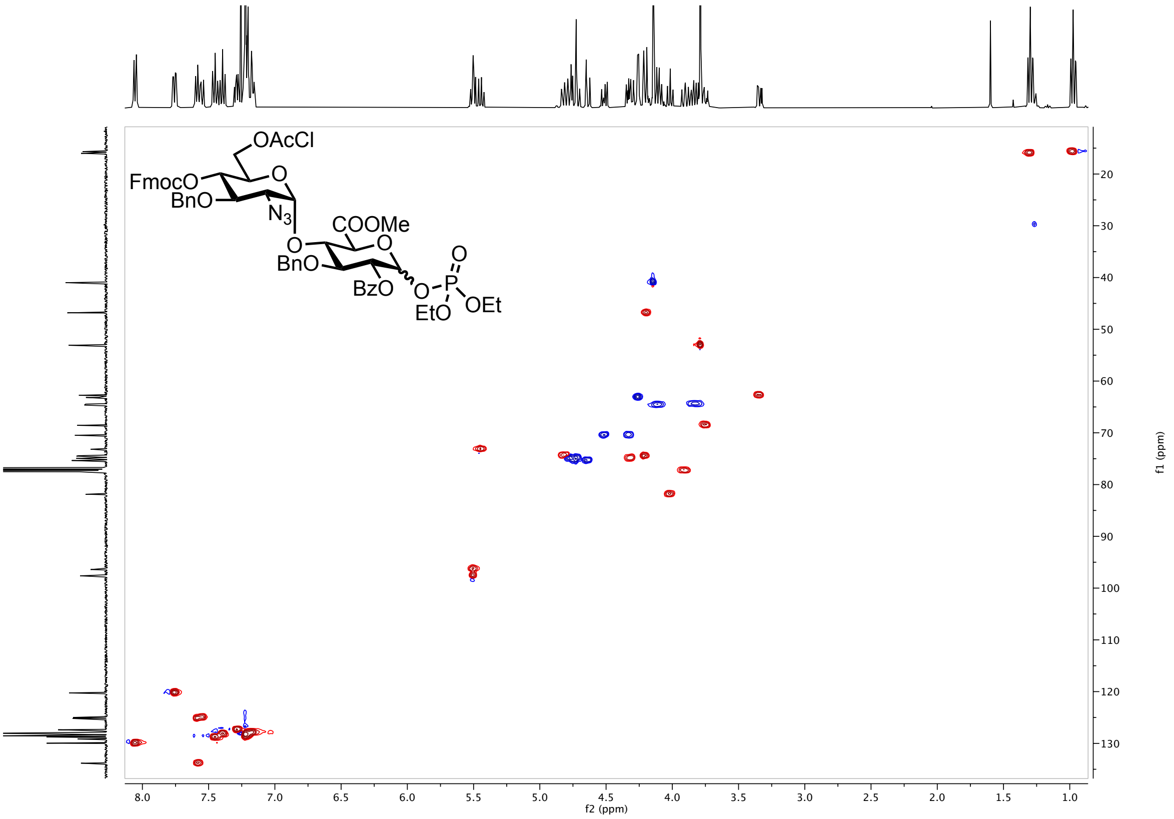

HMBC NMR (400 MHz x 101 MHz, Chloroform-*d*) 6

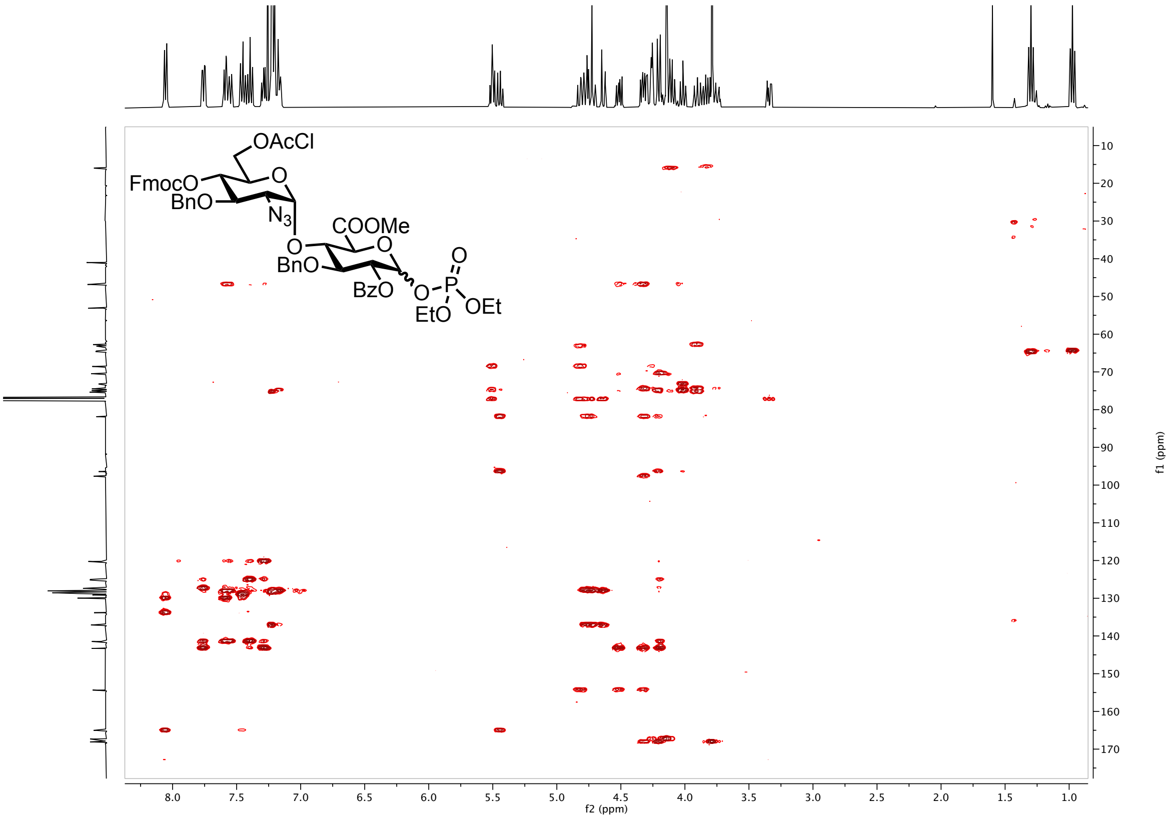

4.5 Compound 9 NMR

^{1}H NMR (600 MHz, Chloroform-*d*) 9

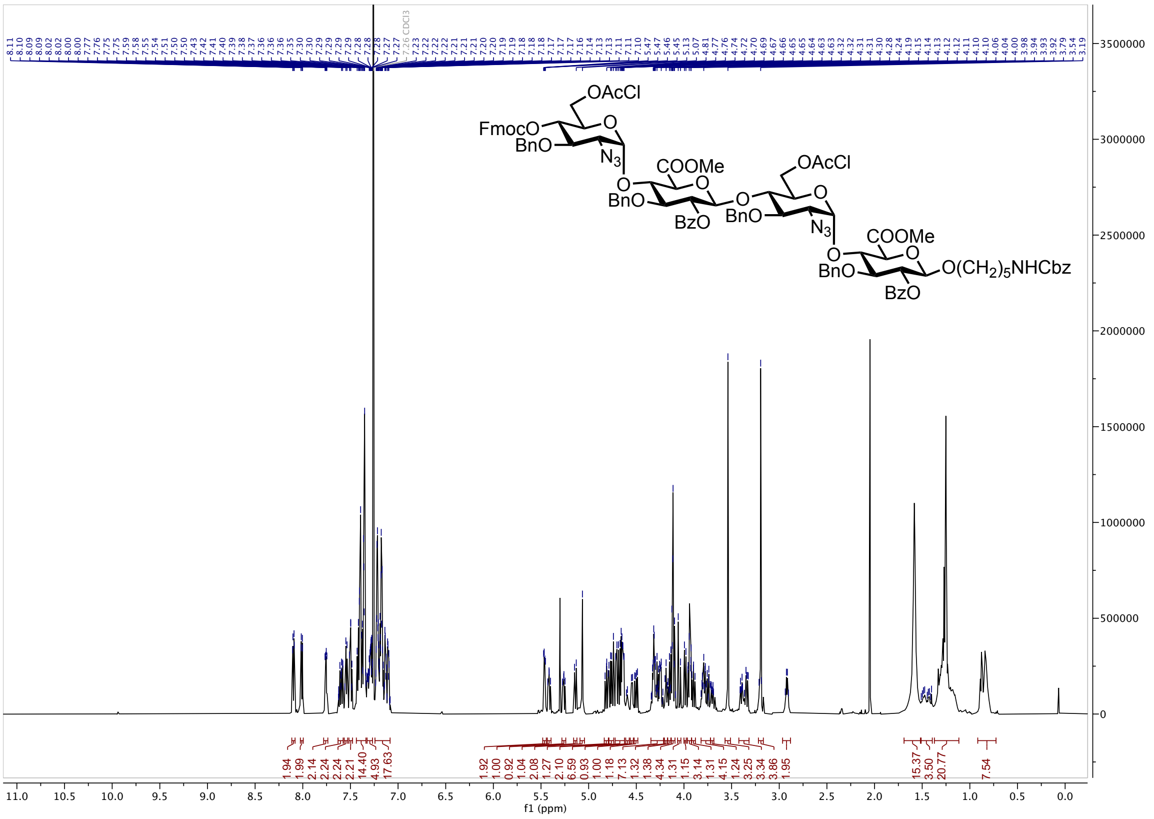

^{13}C NMR (101 MHz, Chloroform-*d*) 9

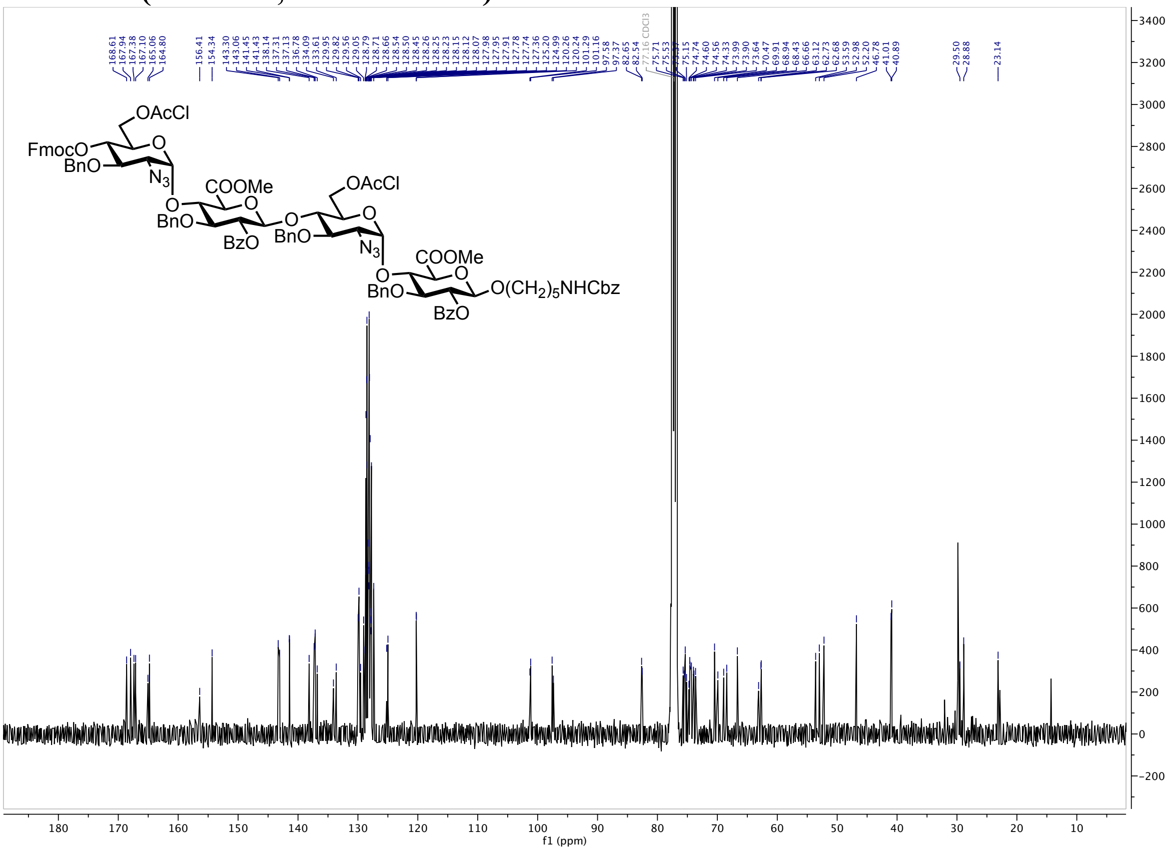

COSY NMR (600 MHz, Chloroform-*d*) 9

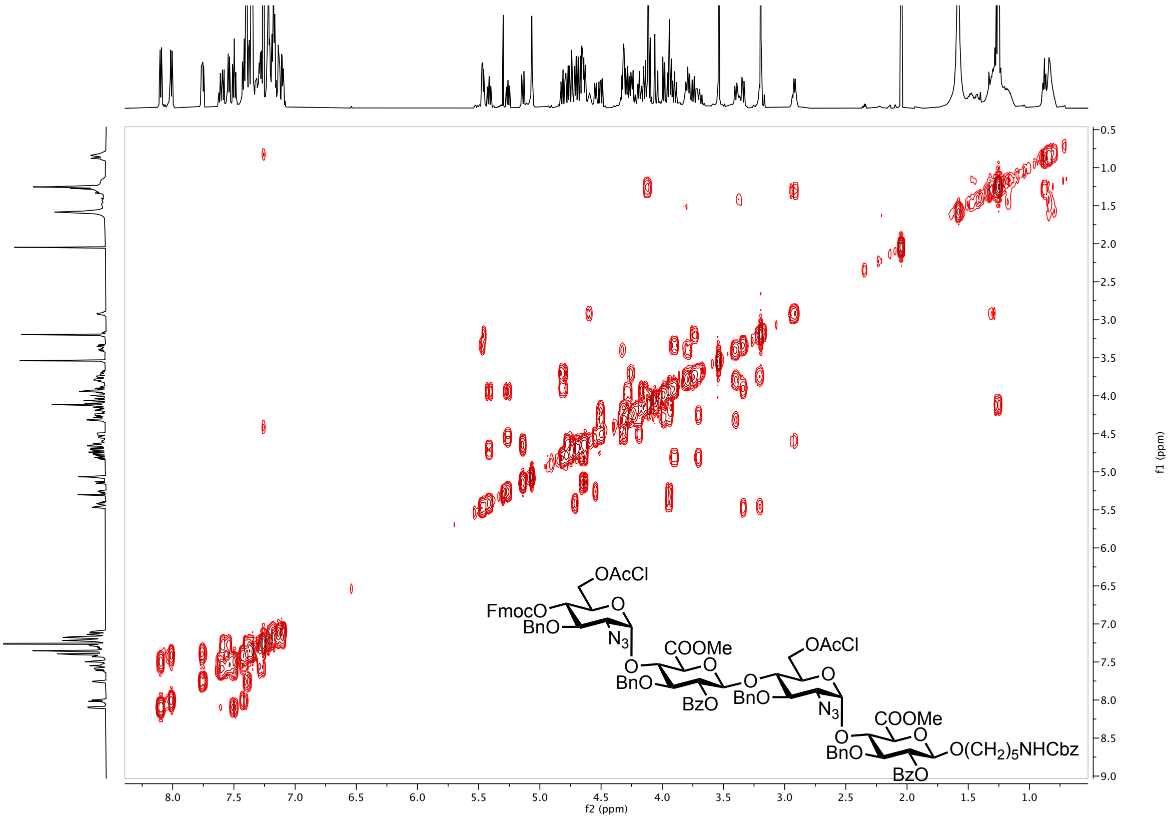

HSQC NMR (600 MHz x 151 MHz, Chloroform-*d*) 9

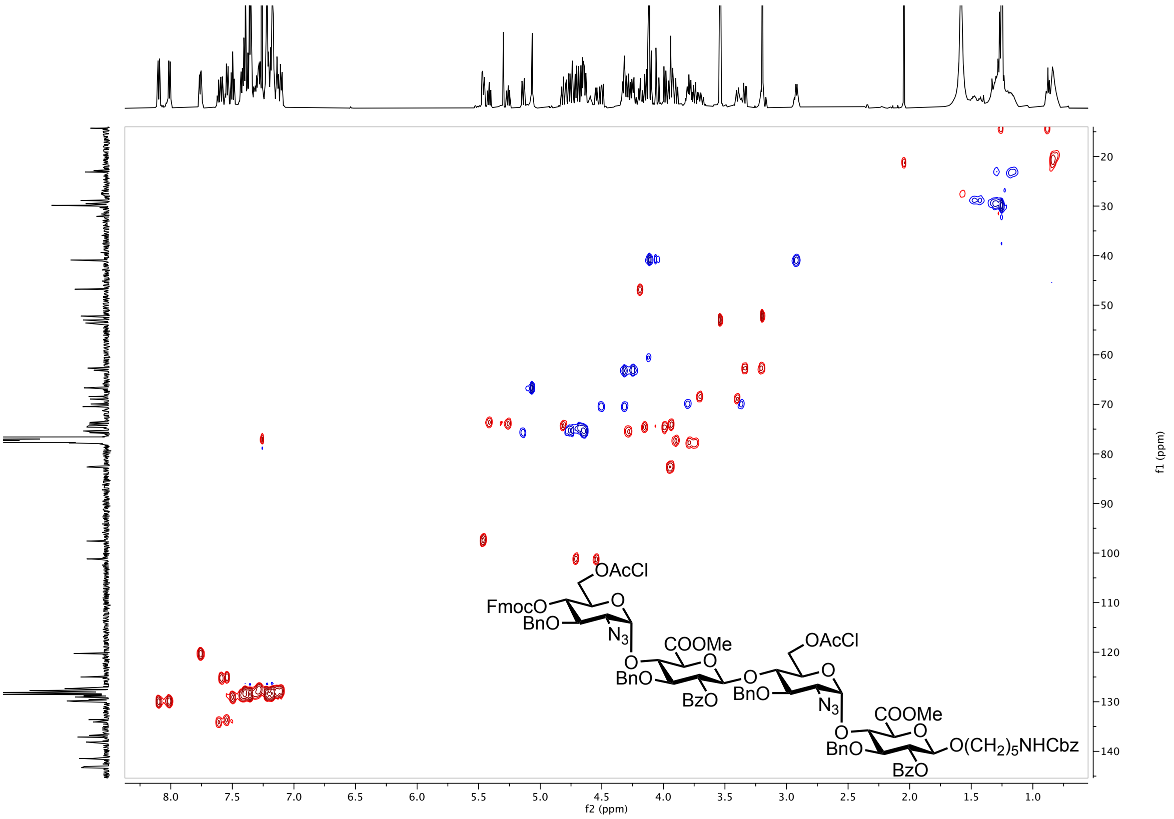

Coupled HSQC NMR (400 MHz x 101 MHz, Chloroform-*d*) 9

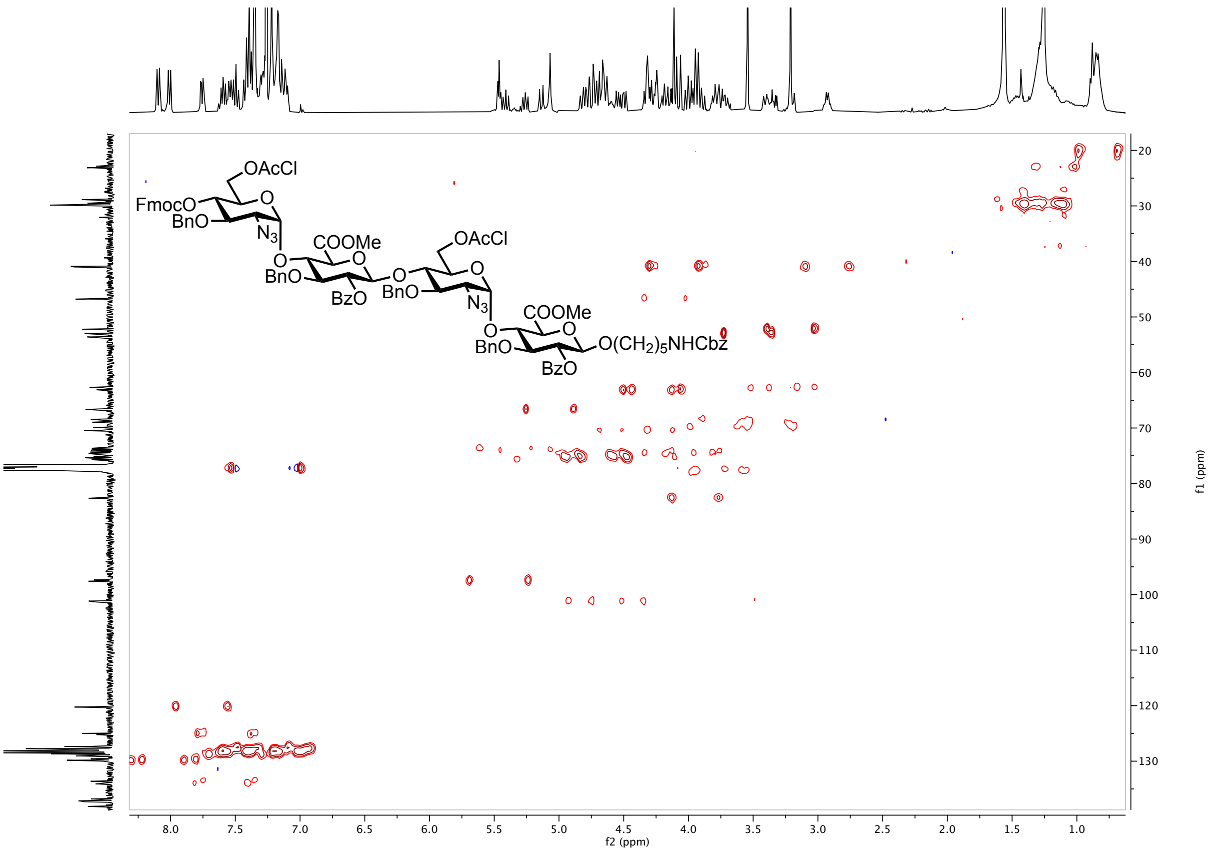

HMBC NMR (400 MHz x 101 MHz, Chloroform-*d*) 9

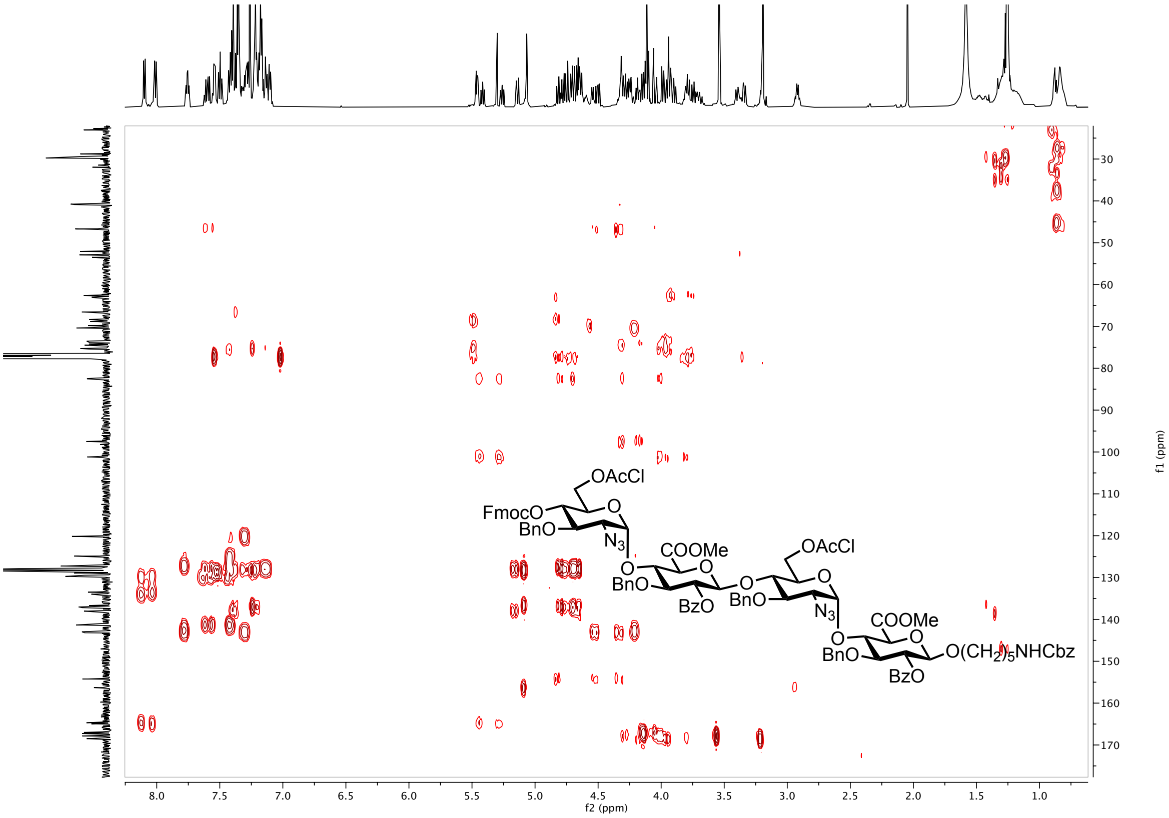

4.6 Compound 10 NMR

^{1}H NMR (400 MHz, Chloroform-*d*) 10

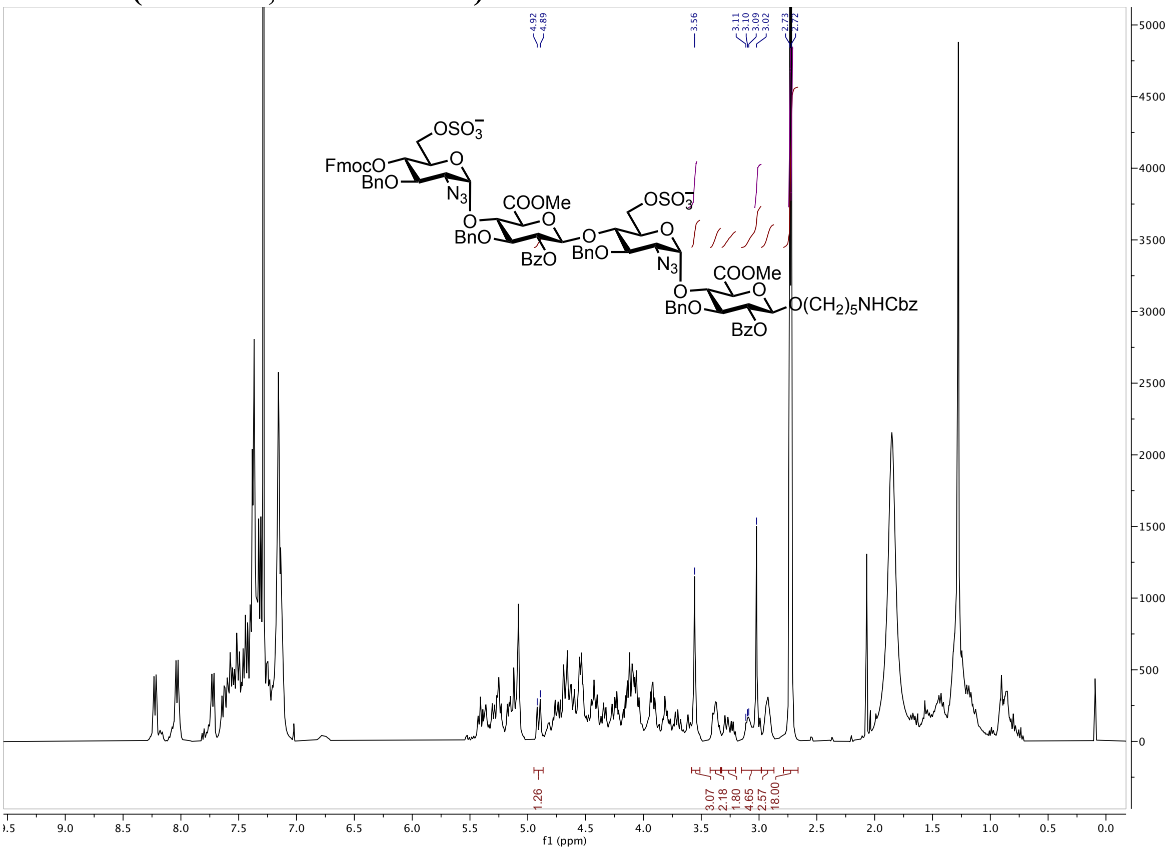

5. References

1 A. B. Pangborn, M. A. Giardello, R. H. Grubbs, R. K. Rosen and F. J. Timmers, *Organometallics*, 1996, **15**, 1518–1520.

2 I. Pongener and G. J. Miller, *J. Org. Chem.*, 2023, **88**, 11130−11139.

3 S. Eller, M. Collot, J. Yin, H. S. Hahm and P. H. Seeberger, *Angew. Chem. Int. Ed.*, 2013, **52**, 5858–5861.
